# Harness High-Temperature Thermal Energy via Elastic Thermoelectric Aerogels

**DOI:** 10.1007/s40820-024-01370-z

**Published:** 2024-03-11

**Authors:** Hongxiong Li, Zhaofu Ding, Quan Zhou, Jun Chen, Zhuoxin Liu, Chunyu Du, Lirong Liang, Guangming Chen

**Affiliations:** 1 College of Materials Science and Engineering, Shenzhen University, Shenzhen, 518055 People’s Republic of China; 2 Department of Bioengineering, University of California, Los Angeles, CA 90095 USA

**Keywords:** Thermoelectrics, Aerogel, Self-powered, High-temperature monitoring, High-temperature warning

## Abstract

Despite notable progress in thermoelectric (TE) materials and devices, developing TE aerogels with high-temperature resistance, superior TE performance and excellent elasticity to enable self-powered high-temperature monitoring/warning in industrial and wearable applications remains a great challenge. Herein, a highly elastic, flame-retardant and high-temperature-resistant TE aerogel, made of poly(3,4-ethylenedioxythiophene):poly(styrenesulfonate)/single-walled carbon nanotube (PEDOT:PSS/SWCNT) composites, has been fabricated, displaying attractive compression-induced power factor enhancement. The as-fabricated sensors with the aerogel can achieve accurately pressure stimuli detection and wide temperature range monitoring. Subsequently, a flexible TE generator is assembled, consisting of 25 aerogels connected in series, capable of delivering a maximum output power of 400 μW when subjected to a temperature difference of 300 K. This demonstrates its outstanding high-temperature heat harvesting capability and promising application prospects for real-time temperature monitoring on industrial high-temperature pipelines. Moreover, the designed self-powered wearable sensing glove can realize precise wide-range temperature detection, high-temperature warning and accurate recognition of human hand gestures. The aerogel-based intelligent wearable sensing system developed for firefighters demonstrates the desired self-powered and highly sensitive high-temperature fire warning capability. Benefitting from these desirable properties, the elastic and high-temperature-resistant aerogels present various promising applications including self-powered high-temperature monitoring, industrial overheat warning, waste heat energy recycling and even wearable healthcare.

**Supplementary Information:**

The online version contains supplementary material available at 10.1007/s40820-024-01370-z.

## Highlights

- A thermoelectric aerogel of highly elastic, flame-retardant and high-temperature-resistant PEDOT:PSS/SWCNT composite is fabricated.
- The assembled thermoelectric generator generates a maximum output power of 400 μW at a temperature difference of 300 K.
- The self-powered wearable sensing glove can achieve wide-range temperature detection, complex hand motion recognition and high-temperature warning.
- The intelligent fire warning system enables highly sensitive and repeatable monitoring and alarm capabilities for high-temperature fire sources.

**Supplementary Information:**

The online version contains supplementary material available at 10.1007/s40820-024-01370-z.

## Graphical abstract

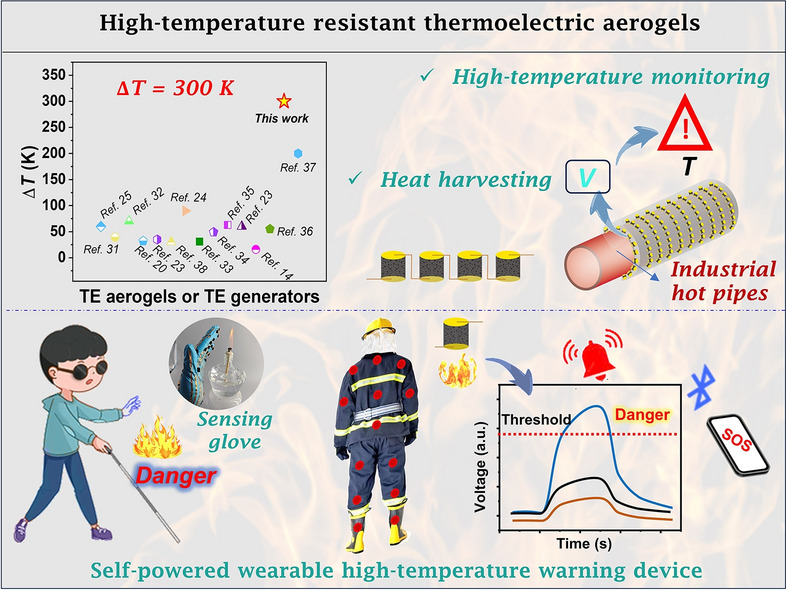

## Introduction

The advent of the Internet of Things (IoT) for industrial management has spurred the demand for intelligent sensors that could be harnessed for high-temperature monitoring/warning to preemptively mitigate potential safety risks and equipment failures. High-temperature environments can pose potential safety risks and equipment failures, making early detection and appropriate action crucial for preventing accidents and ensuring the safety of personnel and equipment. However, traditional high-temperature monitoring and warning systems face challenges such as unstable energy supply, complex wiring requirements and maintenance difficulties [1, 2]. A ray of hope emerges through the utilization of high-temperature thermoelectric (TE) materials that possess the remarkable ability to directly convert thermal energy into electrical energy [3, 4]. This breakthrough holds the promise of surmounting these challenges. Moreover, with the rapid advancement of IoT technology, integrating high-temperature monitoring/warning systems based on durable TE materials with IoT enables real-time data acquisition, data transmission, remote monitoring and warning functions, providing a novel solution for safety management in high-temperature environments.

Given the unique structural advantages (such as three-dimensional porous network structure, light weight and large specific surface area) and the impressive low thermal conductivity of organic aerogels, aerogel-based TE materials are attracting more and more attentions [5–8]. Their TE performance mainly depends on a dimensionless figure of merit, ZT = *S*^2^*σT*/*κ*, where *S*, *σ*, *κ*, *T* and *S*^2^*σ* are the Seebeck coefficient, electrical conductivity, thermal conductivity, absolute temperature and power factor (PF = *S*^2^*σ*), respectively [9, 10]. Various TE aerogels have been prepared using a wide range of materials, including polymers such as poly(3,4-ethylenedioxythiophene):poly(styrenesulfonate) (PEDOT:PSS), polyaniline (PANI) [11, 12] and some insulating polymers (cellulose or polyurethane), carbon-based materials like graphene and carbon nanotubes [8, 13, 14], as well as their composite and hybrid counterparts [15–18]. Among them, polymer/carbon nanotube composite TE aerogels possess high electrical conductivity, excellent structural and mechanical properties, providing great potential in mechanically deformable TE materials and devices. For instance, a low *κ* of 0.055 W m^−1^ K^−1^ and highly elastic single-walled carbon nanotube/polyurethane (SWCNT/PU) TE sponge was obtained by a convenient dipping–drying process, which led to a PF value of 3.87 nW m^−1^ K^−2^ at room temperature [19]. Moreover, their TE properties revealed a unique pressure-driven response, generating an enhanced PF of 11.01 nW m^−1^ K^−2^ at compression strain of 80%. Yao et al. [12] successfully obtained elastic PANI/graphene composite aerogels with an ultrahigh PF value of 20.3 μW m^−1^ K^−2^ (at 127 °C) by *in-situ* oxidative polymerization of aniline on three-dimensional graphene. Despite the reported advancements in various TE aerogel materials, the development of high-temperature-resistant TE aerogels and their performance for broader applications in the future remain highly challenging.

Leveraging the compressibility, conductivity, Seebeck effect and exceptional heat-to-electricity conversion capability of TE aerogels, they find versatile applications as piezoresistive pressure/strain sensors, temperature sensors or TE generators [7, 20–24]. For the realization of these functional applications, temperature is a critical parameter that not only affects the TE properties of the aerogel itself but also determines its performance and practical application as a sensor or generator. Cho et al. [19] developed an elastic SWCNT/PU sponged-based TE generator with 8 p-n couples, generating a large output power of 2.09 μW at a temperature difference (Δ*T*) of 55 K under compressive strain of 80%. Based on the TE mechanism of polydimethylsiloxane-supported PANI/CNT composite aerogel (17.1 μV K^−1^), it can achieve temperature sensing within a Δ*T* of 100 K [21]. Considering the human body as a constant temperature heat source and the demand for self-powered wearable electronic devices, some progress has been made in aerogel-based self-powered wearable temperature sensors, pressure sensors and TE generators [23–26]. For instance, Zhang et al. [26] realized a self-powered wearable temperature–pressure dual-parameter sensors using microstructure PU-supported PEDOT:PSS. However, the reported usage or testing temperatures of organic aerogel-based temperature sensors or TE generators are mostly below 150 °C, and studies to further develop excellent high-temperature-resistant TE aerogels and explore their applications in high-temperature scenarios are still needed to promote practical applications. Therefore, the development of TE aerogels with a wide temperature range application is not only capable of collecting human body heat or high-temperature heat energy, but also suitable for wide-range temperature detection, high-temperature monitoring/warning in industrial and wearable applications.

Herein, highly elastic, flame-retardant and high-temperature-resistant PEDOT:PSS/SWCNT TE aerogels are successfully fabricated according to a convenient solvent displacement, solution mixing and subsequent freeze-drying process, exhibiting a large *S* of 38.9 μV K^−1^ and a low *κ* of 0.074 W m^−1^ K^−1^ at room temperature. The influence of compression strain on their TE properties is then investigated. The composite aerogel not only accurately detects pressure signals but also achieves sensitive temperature sensing over a wide temperature range (25 –325 °C). It can also monitor the temperature of the hot plate continuously heated by the alcohol lamp. Moreover, the assembled TE generator consisting of 25 composite aerogels also demonstrates excellent high-temperature resistance and efficient heat-to-electricity conversion capability, generating a large and stable output power of 400 μW at Δ*T* of 300 K. We also designed an aerogel-based sensing glove to further explore their application as self-powered wide-range temperature detection and complex hand motions recognition. Finally, a self-powered intelligent wearable fire waring system based on aerogels is developed, aiming to investigate its potential application as a high-temperature fire alarm for firefighters. The novel high-temperature-resistant PEDOT:PSS/SWCNT aerogel TE material is promising for applications in wide temperature range heat harvesting, self-powered high-temperature monitoring/warning, and energy-autonomous wearable electronics.

## Experimental Section

### Materials

SWCNT (purity > 90.0 wt%, diameter of 1–3 nm) was obtained from Shenzhen Nanotech Port Co. Ltd, China. PEDOT:PSS aqueous dispersion (Clevios PH1000) with a solid content of 1.0–1.3 wt% was purchased from Heraeus Materials Technology Shanghai Ltd. Nanofibrillated cellulose (NFC, Carboxymethylated, 0.5 wt%) and 3-glycidyloxypropyltrimethoxysilane (GOPS, A.R., 97%) were purchased from Shanghai Aladdin. All other reagents, such as anhydrous ethanol (A.R.) and deionized water, were utilized as received without undergoing additional purification or treatment. Polytetrafluoroethylene (PTFE) molds were bought from Hubei Yongxing Photoelectric Technology Co., Ltd. High-temperature-resistant aluminum silicate ceramic fiber felt was purchased from Weifang Huawei Thermal Insulation Material Co. Ltd, China.

### Preparation of PEDOT:PSS/SWCNT Composite Aerogel

The PEDOT:PSS/SWCNT composite aerogels were prepared by a convenient solution mixing with the assistance of solvent displacement and followed by a vacuum freeze-drying method. Typically, a certain amount of flocculent SWCNT was homogeneously dispersed in ethanol solution with probe ultrasonication for 60 min. Then, with the aid of vacuum filtration, the solvent displacement of ethanol into aqueous solution was realized to obtain the SWCNT aqueous dispersion. Subsequently, a certain amount of NFC was added to the above SWCNT aqueous dispersion for another probe ultrasonication 50 min. Afterward, the PEDOT:PSS aqueous solution was added into the above dispersion and the mixture was ultrasonicated for another 30 min. The crosslink agent GOPS was then dropped into the above dispersion and stirred for 15 min. After that, the PEDOT:PSS/SWCNT aqueous dispersion was drooped into a PTFE mold. Finally, the composite aerogels were obtained after vacuum freeze-drying for 48 h. It should be noted that the weight ratio of PEDOT:PSS:SWCNT:NFC:GOPS was 15:136:15:150. This ratio was determined based on the TE and mechanical properties exhibited by the prepared aerogel, and the corresponding exploration process is shown in Fig. S1 and Table S1. By adjusting the size and shape of the PTFE mold, we can obtain different sizes and shapes of aerogels.

### Preparation of Temperature–Pressure Dual Sensing Glove Based on Aerogels

By utilizing a specific-sized PTFE mold (Fig. S2a), cylindrical PEDOT:PSS/SWCNT composite aerogels with a diameter (*φ*) of 7 mm and a height (*h*) of 5 mm were prepared. These aerogels were subsequently positioned on the knuckle areas of the glove and connected via copper wire based on the memristor principle. To ensure good contact, they were coated with conductive silver glue and copper foil at both hot and cold ends, and finally secured onto the glove's knuckles.

### Fabrication of Aerogel-Based TE Generator

PEDOT:PSS/SWCNT composite aerogels (*φ* = 7 mm, *h* = 10 mm) were prepared with the assistance of a specific-sized PTFE mold (Fig. S2b). Subsequently, the 25 as-fabricated aerogels were interconnected in series through the use of copper wires and silver paste, with copper foils serving as the electrodes at each junction. To provide a stable foundation, temperature-resistant silicone adhesive was employed as substrate.

### Design of Aerogel-Based Intelligent Wearable Sensing System

The prepared aerogels (*φ* = 7 mm, *h* = 5 mm) were distributed within the comfort layer and thermal insulation layer of the fire-resistant clothing, with the top electrode positioned in close proximity to the fire-retardant layer. The designed intelligent sensing system comprises TE aerogels, amplifiers, ADC (Analog-to-Digital Converter), buzzer and Bluetooth module.

### Characterization of Morphology, Structure, Mechanical Property and TE Performance

The micromorphology observations of all the aerogels were conducted using a field-emission scanning electronic microscope (FESEM, Thermo APREO SEM) with an acceleration voltage of 5 kV. Before SEM observation, a thin layer of platinum is plated on the surface of the sample by sputtering. The porosity and pore diameter distribution of the aerogel were determined via mercury injection (AutoPore IV 9500 Version 2.03.01, Micromeritics Instrument Corporation). Raman spectra were acquired on a Raman spectrometer (LabRAM HR Evolution), utilizing a laser with excitation wavelength of 532 nm. Thermogravimetric analysis (TGA) was conducted using a thermal analysis system (TA TGA-Q50), with heating performed at a rate of 10 K min^−1^ over the temperature range of 323 K to 1023 K. The cyclic compressive stress–strain measurement was conducted on a mechanical tensile instrument (EUT4103) with 2 mm min^−1^ speed. For TE performance measurements, the electrical conductivity and Seebeck coefficient of the aerogels under different compression strains were measured using a self-established apparatus (Fig. S3a). The applied compression strain was conducted on a mechanical tensile instrument (EUT4103). A data collection device (Keithley 2700) was used to record resistance values, which were then combined with the size of the aerogel to obtain electrical conductivity values. For controlling the Δ*T* applied during the measurement of the Seebeck coefficient, a constant-temperature water bath (DCW-3006) was utilized when Δ*T* is below 100 K, while a high-temperature control system (YuDian PID control system) was employed when Δ*T* exceeded 100 K. For the experiment of the relationship between the generated voltage and Δ*T*, we conducted measurements on at least five samples, and the average value was utilized for analysis. The error bars for the above TE tests were determined based on the average value of at least five test results and their corresponding standard deviation. The thermal conductivity of the aerogels was recorded on a commercial thermal conductivity tester instrument (Hot Disk TPS3500). For the output performance measurements, the output voltage, output current and electrical resistance were collected using a self-established apparatus (as shown in Fig. S3b), which consists of a temperature control system (YuDian PID control system), a data acquisition system (Keithley 2700), a hot plate and a cold plate.

## Results and Discussion

### Elastic Aerogels of PEDOT:PSS/SWCNT Composites

**Fig. 1:**
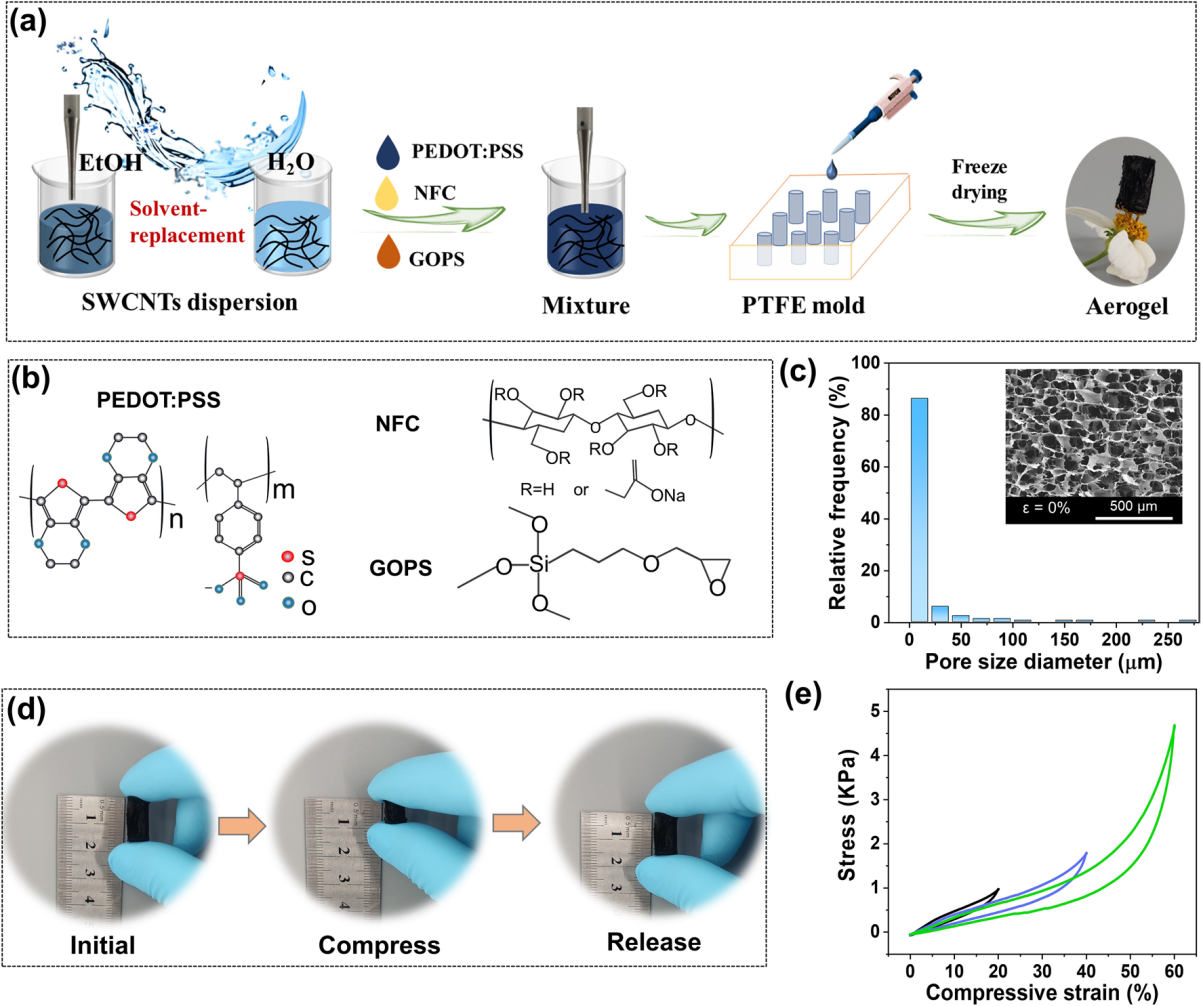
Aerogel preparation and mechanical properties evaluation. **a** Schematic illustration of the preparation process of the elastic PEDOT:PSS/SWCNT composite aerogel. **b** Chemical structures of PEDOT:PSS, NFC and GOPS. **c** Pore size diameter distribution of aerogel based on mercury injection measurement. Inset of Fig. shows the SEM image of the aerogel. **d** The digital images depict the states of the aerogel: before (left), during (middle), and after (right) finger pressing. **e** Compressive stress–strain curves of TE aerogel at different compressive stains

The elastic, light-weight and porous aerogels of PEDOT:PSS/SWCNT composites were successfully fabricated via a convenient solvent displacement, solution mixing and subsequent freeze-drying process, as shown in Fig. 1a. The formation mechanism mainly attributes to the uniform dispersion of SWCNT through solvent displacement and the formation of an interconnected three-dimensional porous network resulting from the interactions of various components (Fig. 1b) and freeze-drying process. Moreover, composite aerogels with adjustable sizes and shapes can be readily achieved by employing corresponding polytetrafluoroethylene (PTFE) molds. As reflected in Fig. 1c, the original aerogel displays a typical lamellar and interconnected porous structure constructed with “layer-strut” skeletons, where more than 85% of the pore size diameter distribution of aerogel are distributed between 5 and 25 μm. Such high and uniform porosity (measured porosity: 87%) of the aerogel results in an ultralight capability (0.01 g cm^−3^) that can easily stand on the stamen of a flower without exhibiting noticeable deformation or damage (Fig. 1a) and a desired low thermal conductivity (*k* = 0.074 W m^−1^ K^−1^). Besides, the composite aerogel exhibits super mechanical elasticity, which is confirmed by finger compression cycles (Fig. 1d). Its superstability and recoverability are further demonstrated by the compressive stress–strain tests, in which the maximum stress value can be well maintained after subjecting the aerogel to repeated compression cycles with varying strains (20%–80%), as depicted in Figs. 1e and S4.

### Structure and TE Performance of PEDOT:PSS/SWCNT Composite Aerogel

**Fig. 2:**
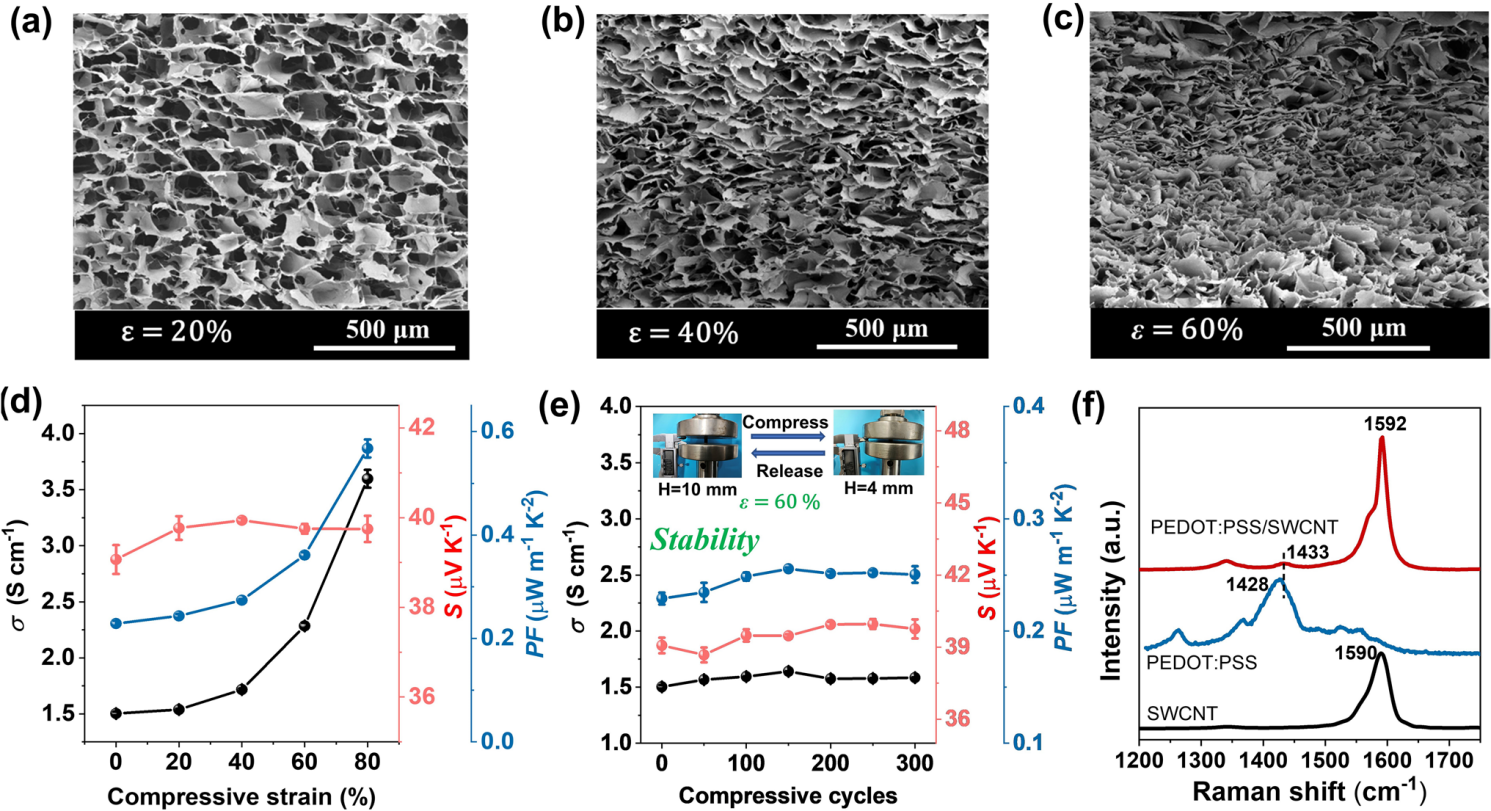
Microstructure and TE properties of the aerogels. **a–c** SEM images of TE composite aerogel under different compression strains ((a) *ε* = 20%, (b) *ε* = 40%, (c) *ε* = 60%). **d** The TE properties variations when subjected to compression strain (0–80%). **e** The variation in TE properties for the aerogel after multiple compression cycles with 60% strain. **f** Raman spectra of the pure SWCNT, PEDOT:PSS and the composite aerogel

It is well established that the porous microstructure has notable influence on the mechanical and TE properties of the resultant aerogels. As revealed by the SEM images in Fig. 2a–c, with the increase in compression strain (*ε*), the original pores composed of “layer-strut” skeletons are distorted and the layer spaces are reduced, resulting in the decrease in porosity. For the pressure-driven TE properties (Fig. 2d), note that the *σ* slightly increases in the lower compression strain (less than 40%) and then notably enhances in the subsequent strain range (40%–80%); these variations highly depend on the structural evolution of pores and the reduction in porosity during compressing. A largest *σ* of 3.7 S cm^−1^ is obtained at compression strain of 80%, the great enhancement mainly attributes to the generation of the carrier conductive transport pathways and good electrical contact between the intrinsic conductive skeleton layers induced by distorted and compressive pores. Undoubtedly, the decrease in porosity also leads to an increase in thermal conductivity, *i.e.*, the *k* also increases from 0.074 to 0.167 W m^−1^ K^−1^ with increased strain from 0 to 50%. The *k* value is decided by the formula *κ* = *ρ* × *c*_*v*_ × *α,* where* ρ*, *c*_*v*_ and *α* are density, volumetric heat capacity and thermal diffusion coefficient, respectively. The corresponding experimental results are shown in Figs. S5, S6 and Table S2. Besides, the *S* is nearly pressure independent, as most previously reported [19, 25, 31]. As a result, the aerogel reveals a significantly enhanced PF with the increase in compression strain, reaching a maximum value of 0.58 μW m^−1^ K^−2^ at the strain of 80% (Fig. 2d). Compared with the room temperature TE properties of conducting polymers, carbon nanoparticles or their composite aerogels reported in various literatures (Table S3), the PEDOT:PSS/SWCNT composite aerogels obtained in this work reveal desired TE performance. More importantly, as shown in Fig. 2e, relatively stable *σ* and *S* can still be maintained after repeated compression cycles (0–300) with 60% strain, indicating their excellent mechanically stable TE properties and high durability. The high and stable TE properties also originate from the effective interfacial *π*-*π* interactions and partial charge carries transport between PEDOT:PSS molecular chains and SWCNT, as demonstrated by Raman analysis (Fig. 2f). As shown, the pristine PEDOT:PSS exhibits three characteristic peaks at 1364, 1428 and 1511 cm^−1^, which are attributed to the asymmetric C–C stretching vibrations, symmetric C = C stretching vibrations and asymmetric C = C stretching vibrations, respectively [27]. The pristine SWCNT displays a typical G band (in-plane stretching E_2g_ mode) at 1590 cm^−1^. For the resultant composite aerogels, the peak intensity of PEDOT:PSS is significantly weakened and almost disappeared, while the peak of SWCNT at 1590 cm^−1^ reveals a notable blue-shift to 1592 cm^−1^ [27, 28]. This observation demonstrates the presence of interfacial *π*-*π* interactions and effective charge transfer between PEDOT molecules and SWCNT [27, 28].

### Performance of PEDOT:PSS/SWCNT Composite Aerogels as A Strain or Temperature Sensor

**Fig. 3:**
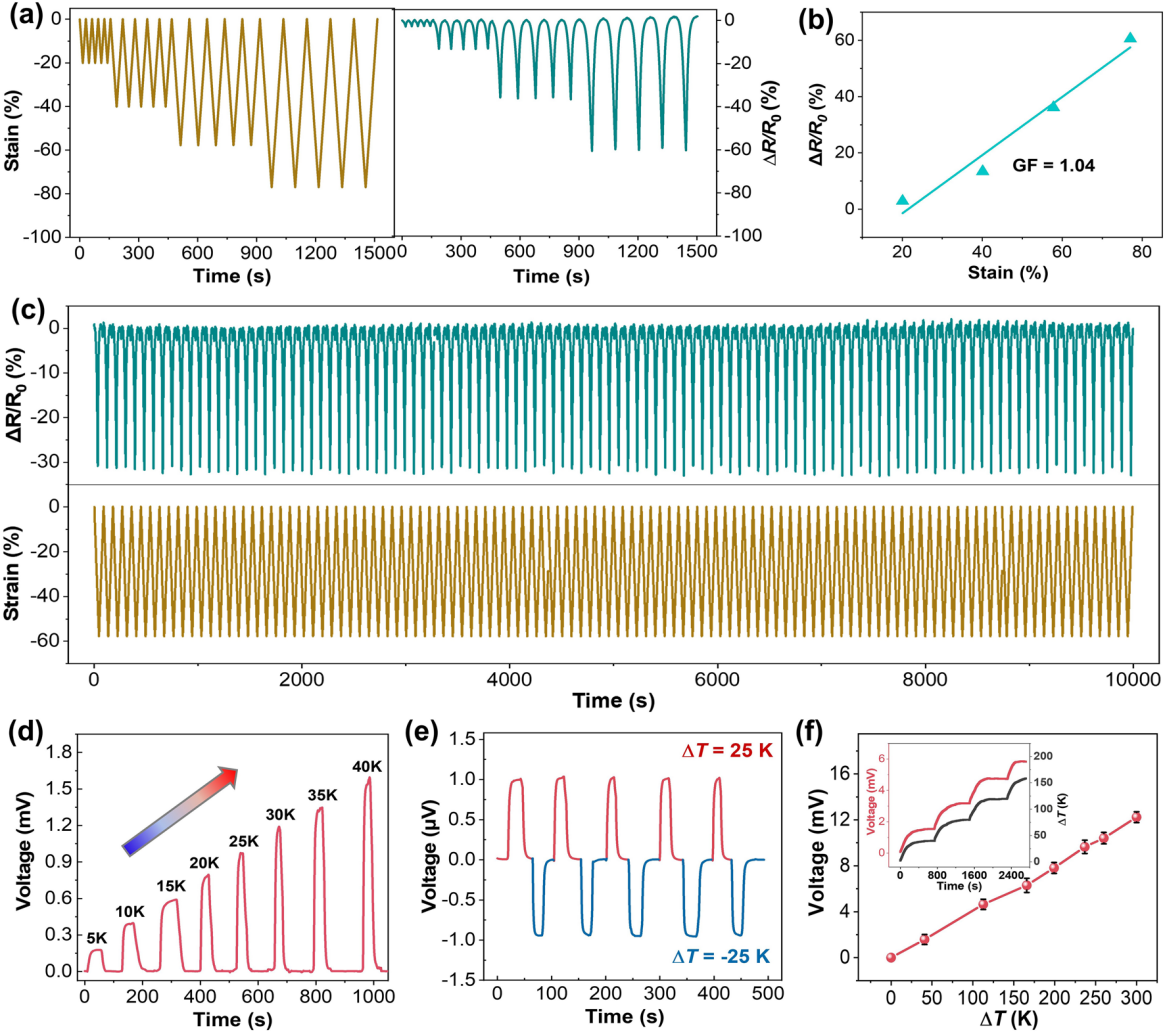
An aerogel-based temperature and pressure dual-mode sensor. **a** Real-time relative resistance change (Δ*R*/*R*_0_) response of the aerogel subjected to various compression strains. **b** The Δ*R*/*R*_0_ of the aerogel as a function of strain. **c** The durability test under cyclic loading of 60% strain. **d** Real-time voltage responses of the aerogel at different Δ*T*. **e** Real-time voltage response of the aerogel under alternating and opposite temperature differences (Δ*T* =  ± 25 K). **f** The generated voltage as a function of Δ*T*, where the inset shows the real-time voltage response to the applied Δ*T*

Given the high elasticity and the desired mechanical durability, the conductive composite aerogels are particularly suitable for strain sensor. Specifically, the construction of 3D interconnected porous microstructure with plenty of junctions and interfaces endows the aerogels with high electrical resistance-strain sensitivity. As shown in Fig. 3a, the aerogel produces stable and repeatable output electrical signals during five consecutive compression–release cycles where the strain is varied from 20% to 80%, demonstrating high and reversible strain sensing ability. As the applied strain increases from 20% to 80%, the corresponding resistance change (Δ*R*/*R*_0_, where *R*_0_, and *R* are the resistance at zero strain and applied strain, respectively) varies from − 5% to − 60%. The decided gauge factor (GF = Δ*R*/*R*_0_) is about 1.04 at a wide strain range of 20%–80% (Fig. 3b), revealing acceptable sensitivity to strain [20, 23, 29]. Besides stability and gauge factor, durability is another significant parameter to evaluate the performance of strain sensor [19, 23, 30]. As reflected in Fig. 3c, the Δ*R*/*R*_0_ exhibits good repeatability and negligible fluctuations over 115 continuous compressing-releasing cycles at a strain of 60%, and such excellent durability is rather advantageous for practical applications.

Considering the large and stable Seebeck coefficient of the composite aerogels, they can be readily used as a temperature sensor for temperature detection, where the electrical signals (thermo-voltage) result from the Seebeck effect of aerogel itself. As illustrated in Fig. 3d, as the applied external temperature difference (Δ*T*) stimuli increases from 5 to 40 K, the real-time voltage linearly increases, confirming that the aerogel sensor can accurately detect the temperature variation. More importantly, the aerogel sensor also exhibits excellent sensing stability and repeatability, as demonstrated by the voltage changes in response to steady and alternating temperature difference (Fig. 3e). At Δ*T* =  ± 25 K, it presents stable voltages of ± 1.0 mV, which are also consistent with the sensitivity data and Seebeck coefficient (Figs. 2d and 3d). Moreover, this aerogel-based sensor exhibits the capability to detect not only lower temperatures but also effectively respond to higher temperature variations. As shown in Fig. 3f, as the Δ*T* continues to increase from 50 to 300 K, its corresponding voltage increases linearly, generating a large voltage of 12.2 mV at Δ*T* of 300 K. The inset in Fig. 3f shows the real-time voltage variation when continuous Δ*T* is applied. As shown, the voltage varies synchronously with the Δ*T*, indicating its fast and sensitive responsiveness. When the aerogel is placed on the hot plate heated by an alcohol lamp, it can also produce a large and stable voltage of ~ 7.2 mV (Fig. S7). Such excellent sensitivity for both low- and high-temperature gradients detection is mainly attributed to the following two aspects: the high and stable TE properties of the composite aerogel, and the desired high-temperature stability. For the former, Seebeck coefficient is the key parameter to decide the sensitivity of temperature sensor. For the latter, the temperature stability of the composite aerogel is confirmed by thermogravimetric analysis (TGA). As illustrated in Fig. S8a, at high temperature of 350 °C, the weight loss of the composite aerogel is only 9%, indicating its excellent high-temperature stability, which is sufficient to achieve the above-mentioned sensing performance at Δ*T* of 300 K. This stability is mainly due to the presence of SWCNT with stable structure and properties, whose weight content can remain 85% at a high temperature of 700 °C (Fig. S8b).

### High-Temperature-Resistant Aerogel-Based TE Generator

**Fig. 4:**
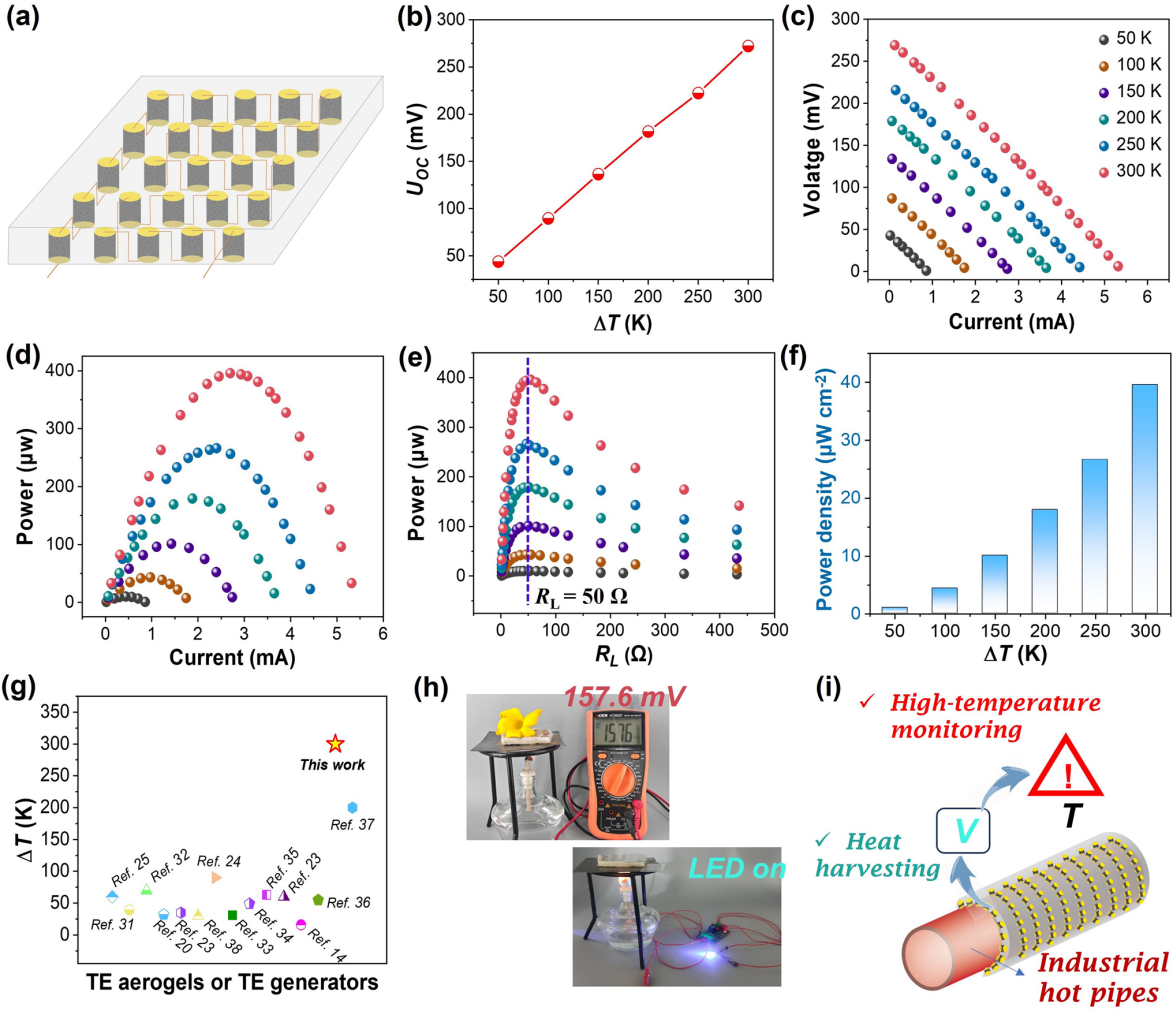
An aerogel-based TE generator. **a** Illustration showing the TE generator consisting of 25 aerogels in series. **b** Open-circuit voltages generated by the aerogel-based TE generator under different Δ*T*. **c** The relationship between the generated output voltage as a function of circuit current at different Δ*T*. Output power of TE generator as a function of **d** circuit current and **e** external load resistance under different Δ*T*. **f** The power density of the TE generator at different Δ*T*. **g** The comparison of the highest testing temperatures for most TE aerogels and their generators reported in the literature. **h** Photographs showing the generated voltage or powering a LED by harvesting alcohol lamp flame heat energy. **i** Illustration showing the high-temperature application scenario of the aerogel-based TE generator by utilizing heat energy on industrial hot pipes

It is well established that the output performance of the TE generators highly depends on the TE properties of TE legs, the number of TE legs (*N*) and the temperature difference (Δ*T*). Considering the high TE performance and good heat-resistant ability of the PEDOT:PSS/SWCNT composite aerogels, a flexible TE generator with 25 aerogels connected in series is subsequently designed and fabricated, as shown in Fig. 4a. Perforated aluminum silicate ceramic fiber felt is used as a substrate for the TE generator because of its flexibility, low thermal conductivity and high-temperature resistance. As presented in Fig. 4b, the as-fabricated TE generator reveals linearly increased open-circuit voltage (*U*_OC_) with increasing Δ*T* from 50 to 300 K, generating a high *U*_OC_ of 275 mV at Δ*T* of 300 K, which matches well with the theoretical value (*U*_OC-theory_ = *N* × *S* × Δ*T,* where *N* is the number of TE aerogels, *S* is the Seebeck coefficient). When various external load resistors (*R*_L_) are connected in series with the TE generator, its practical output performance can be evaluated by the generated output voltage (*U*), current (*I*) and the corresponding output power (*P*_out_ = *I* × *U*) under the desired Δ*T*. As illustrated in Figs. 4c–e and S9, the *U* exhibits a linear relationship with the *I*, while the *P*_out_ shows a parabolic curve relation with either the *I*, *R*_L_ or *U*, which are in accordance with the theoretical output performance. The corresponding *U* and* I* as a function of *R*_L_ are also presented in Fig. S10a, b, respectively. Apparently, both *U* and *P*_out_ are greatly enhanced with increasing the Δ*T* (50–300 K), generating a maximum output power (*P*_max_) of 400 μW at Δ*T* of 300 K. Besides, the corresponding *R*_L_ at *P*_max_ is about 51 Ω, which is consistent with the measured internal resistance (*R*_i_ = 50.4 Ω, Fig. S11) of the TE generator, evidencing that *P*_max_ $\left( {P_{{{\text{out}}}} = \frac{{{{U}}_{{\text{OC }}}^{{2}} {{ R}}_{{\text{L}}} }}{{{{(R}}_{{\text{i}}} {{- R}}_{{\text{L}}} {)}^{{2}} {{ + 4R}}_{{\text{i}}} {{R}}_{{\text{L}}} )}}} \right)$ is achieved when the *R*_L_ is equal to the *R*_i_. The corresponding power densities at different Δ*T* are shown in Fig. 4f, and a maximum power density of 40 μW cm^−2^ is obtained at Δ*T* = 300 K. Compared with the currently reported aerogel-based TE generators, the elastic PEDOT:PSS/SWCNT composite aerogel TE generator developed in this work exhibits notably high output power and power density (Table S4). Figure 4g summarizes the highest testing temperatures for most TE aerogels and their generators reported by literatures [14, 20, 23–25, 30–38]. Obviously, the testing or application temperatures of our fabricated TE aerogel and its generator are significantly higher than that of mostly reported aerogels and their generators (Tables S3, S4). Thus, the as-fabricated composite aerogel and its TE generator reveal excellent temperature resistance and efficient heat-to-electricity conversion capability, suggesting their good adaptability to wide-range temperature scenarios.

To further demonstrate its practical usability, the aerogel-based TE generator is placed on a high-temperature hot plate to harvest the heat energy of the flame. As shown in Video S1 and Fig. 4h, the voltage generated by the TE generator increases in step with the temperature difference during the flame heating the hot plate, revealing a large and stable voltage of ~ 157.6 mV. Moreover, with the assistance of a voltage amplifier, the TE generator can also rapidly light up a LED by utilizing this temperature difference (Fig. 4h). Combining the desired merits of excellent temperature resistance, low thermal conductivity, ultralight weight and high TE properties, this TE generator can also be conveniently wrapped around heat flow or hot steam pipes (Fig. 4i), enabling simultaneous effective heat recovery and heat insulation. Besides, this TE generator could be used as a reliable self-powered high-temperature monitoring device for real-time detection of surface temperature on pipes due to linear relationship between temperature and output voltage. This feature provides a unique advantage in the field of industrial high-temperature monitoring. Thus, with the excellent TE properties and the dimensional and structural tunability of the aerogel, the application scenarios of the resulting aerogel assembled TE generator are expected to expand to multiple fields.

### Self-Powered Wearable Sensing Devices for High-Temperature Monitoring/Warning and Complex Hand Motions Recognition

**Fig. 5:**
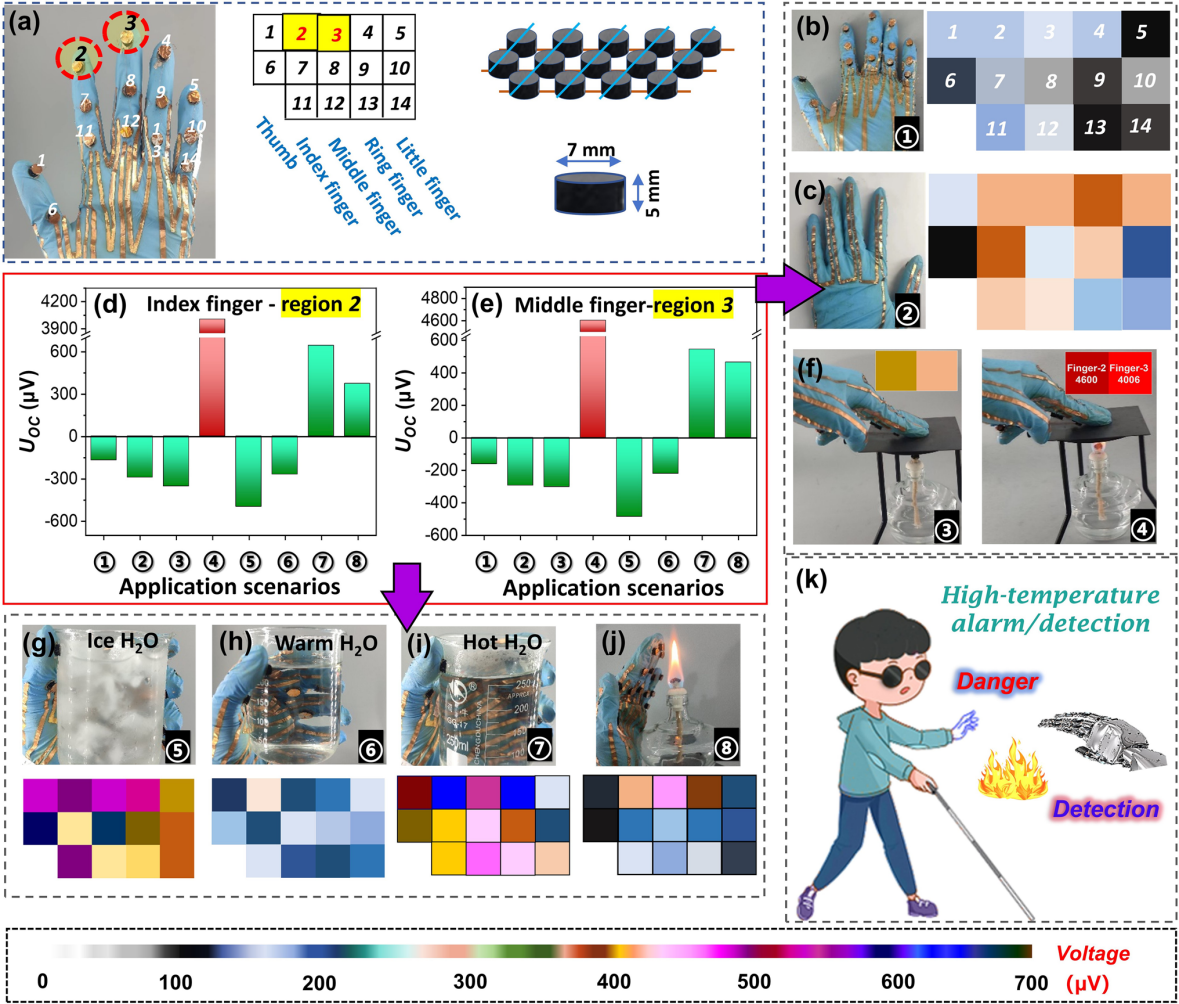
Aerogel-based integrated sensor array and its applications. **a** Photograph showing the flexible self-powered sensing glove and illustration of the glove connection mode. **b** Photograph showing the hand gesture of spread and the corresponding voltage distribution maps at fourteen knuckles on the wearable TE sensing glove. **c** Photograph showing the hand pressing on a tabletop and the corresponding voltage distribution maps. **d, e** The characteristic voltage signals at knuckle 2 and knuckle 3 when different gestures or various objects are touched. **f** Illustration of the fingers pointing at an unheated or heated plate, and the characteristic voltage map at knuckle 2 and knuckle 3. Photographs showing the hand griping a beaker containing** g** ice water, **h** warm water, or **i** hot water, and the corresponding map of voltage signals at the fourteen knuckles. **j** Photograph showing the hand approaching the flame of an alcohol lamp and the corresponding voltage distribution maps. **k** Illustration showing the self-powered application scenario of the composite TE aerogels for blind people or robots high-temperature alarm/detection

The ultralight weight, elastic and high-performance TE aerogels are particularly suitable for wearable sensing devices. In particular, to ensure low cost and wide applicability, a simple device integration method is also highly desired. As shown in Fig. 5a, simple-structural and self-powered wearable sensing glove is constructed with fourteen TE aerogels, where these aerogels were positioned on fourteen knuckles in five fingers and connected via copper wire with copper foil as electrodes based on the memristor principle. The temperature-dependent sensing capability is evaluated by the voltage signals generated from the Seebeck effect of the aerogel itself. Of particular note is that the voltage signals generated at the fourteen knuckles are different due to variations in distance and hand gestures (such as “Spread,” “Point,” “Pinch” or “Grip”). As depicted in Fig. 5b, there is minimal variation in the voltage signals at the fourteen knuckles when the hand is spread. However, when the palm is pressed against a room-temperature tabletop, a significant variation in the voltage signals occurs at the fourteen knuckles due to improved contact between the sensing aerogels and the human skin (Fig. 5c). Figure 5d, e provides characteristic voltage signals at knuckles 2 and 3 when different gestures or various objects are touched. Apparently, when placed on a plate heated by an alcohol lamp, the "point" gesture exhibits extremely high voltage at knuckles 2 and 3, approximately 4600 and 4006 μV, respectively (Fig. 5f). As shown in Fig. 5g–i, when griping a beaker containing ice water, warm water, or hot water, distinct voltage signals are generated at the fourteen knuckles, demonstrating precise temperature perception. Moreover, this integrated sensor demonstrates high sensitivity in perceiving the temperature of flame (Fig. 5j), highlighting its significant potential for applications in assisting visually impaired individuals or robots in sensing high temperatures (Fig. 5k). It is noteworthy that when the finger skin temperature is higher or lower than the temperature of the object being touched, the corresponding voltage signals produced are inversely proportional (Fig. 5d, e), further confirming its sensitive responsiveness.

Besides its precise temperature sensing capability, the integrated sensor glove can also achieve accurate recognition of complex hand gestures (such as spread, point, pinch and grip, as shown in Fig. S12) through the combination of optimal algorithms (Fig. S13), similar to the film-based sensor we previously reported [39]. As shown in Fig. S14, the voltage amplitude variations of the hand gestures of "point," "pinch" and "grip" near the threshold voltage are significantly different, indicating the precise hand gesture recognition capability of the integrated aerogel-based sensor glove.

### Self-Powered Intelligent Wearable Fire Warning System for Firefighters

**Fig. 6:**
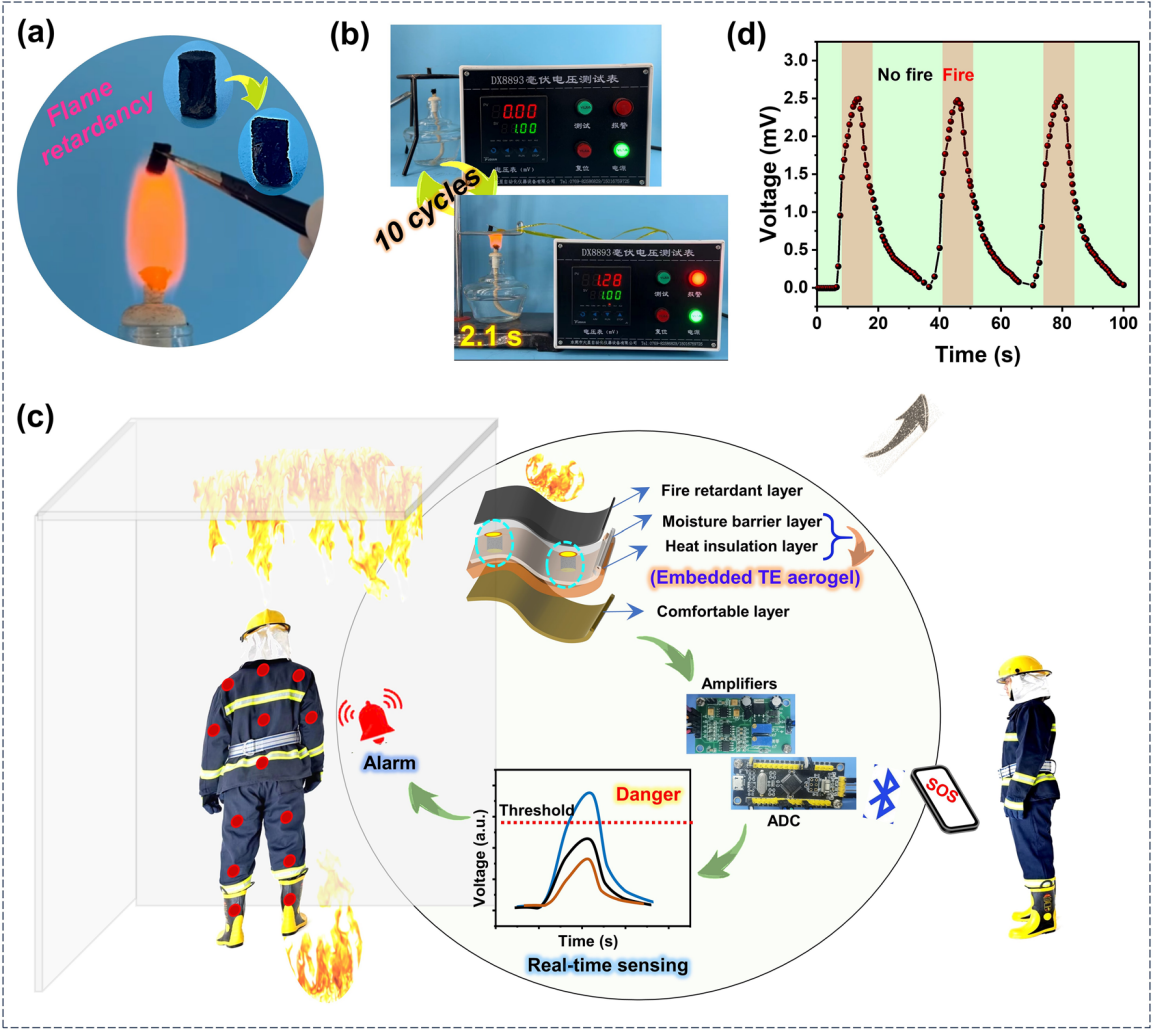
Aerogel-based self-powered fire warning system. **a** Flame retardancy property of the aerogel. **b** Fire warning test for aerogel when exposed to the flame of alcohol lamp. **c** Schematic showing the aerogel-based self-powered wearable fire warning system that provides high-temperature warning to firefighters. **d** Voltage curve of aerogel-based self-powered wearable device in three cyclic fire warning tests

The prepared aerogels exhibit exceptional flame retardance property. As illustrated in Fig. 6a and Video S2, a vertical burning test was conducted to visually assess the flame-retardant performance of the aerogel. It is evident that the aerogel remained unignited and maintained its structural integrity even after being exposed to a flame for 30 s. The unique flame-retardant properties of this aerogel should be primarily attributed to two factors. Firstly, the SWCNT possesses inherent excellent thermal stability (as demonstrated by TGA analysis, Fig. S8). Secondly, the addition of crosslinking agent of GOPS enhances the structural stability and mechanical strength of the aerogel. When exposed to high temperatures, GOPS decomposes and generates smoke-free and non-toxic silicon oxide residues. These residues form a protective layer on the surface of the aerogel, effectively preventing flame propagation and combustion. Considering the practical application scenario, the flame retardancy and excellent heat-to-electricity conversion capability of aerogels position them as promising self-powered fire warning materials. As shown in Fig. 6b, an early fire warning system without need for an external power source is constructed by connecting the aerogel directly to the millivolt voltage alarm. The early warning trigger voltage for the aerogel is set at 1 mV. The corresponding fire warning process is depicted in Fig. 6b and Video S3, providing an intuitive evaluation of its fire warning performance. Remarkably, even after 10 cycles, the aerogel consistently triggers the alarm within 3 s upon exposure to the flame of an alcohol lamp, demonstrating its reliable and repeatable fire warning behavior. The trigger times corresponding to each fire warning during these 10 cycles are shown in Fig. S15. Table S5 summarizes the response time of various fire warning material base on TE materials. Compared with other TE warning materials, the PEDOT:PSS/SWCNT aerogel developed in this work exhibits notably fast fire warning capability, suggesting its high potential in fire warning application.

Furthermore, considering the inherent risks faced by firefighters during firefighting and rescue operations, especially when exposed to extreme temperatures and flames, integrating flame-retardant and highly sensitive temperature-sensing aerogels into firefighting suits emerges as an effective strategy to protect firefighters from burns. This approach could help mitigate the potential dangers that firefighters inevitably encounter, providing enhanced safety during fire suppression and rescue missions. To take full advantage of the prepared aerogels, we conceived a self-powered smart wearable fire warning system to alert firefighters once a fire source approaches the protective clothing. As illustrated in Fig. 6c, the prepared aerogels are integrated into the moisture barrier and thermal insulation layers on the back of the firefighting suit, with the electrodes on top of the aerogel tightly contacting the flame-retardant layer to achieve more effective thermal-to-electrical conversion. Besides, the TE aerogels are strategically distributed across the back, waist and legs of the firefighting cloth to effectively monitor fire sources from different directions. The intelligent fire warning system is composed of energy collection (TE aerogels), amplifier, ADC (Analog-to-Digital Converter), buzzer and Bluetooth module (Fig. S16). To confirm the usability of the designed system in a fire scenario, we test this system with open flames indoors. As shown in Video S4, when no flames are in proximity, everything remains calm and undisturbed. However, once a flame is detected and reaches the default threshold, the alarm system is immediately triggered, activating an alert to remind firefighters to stay away from the fire source or evacuate safely. Simultaneously, a Bluetooth module will transmit the information in real-time to a smartphone or computer, allowing other firefighters to monitor the personal safety of the current firefighter and provide timely support. As shown in Fig. 6d, the self-powered fire warning system, designed to intermittently expose the alcohol lamp flame at 30 s cycle periods, consistently generates an average output voltage of 2.48 mV with a small standard deviation of 2.2%. This indicates the reliable repeatability of the aerogel-based fire warning system. Therefore, the developed intelligent wearable sensing system, based on high-temperature-resistant TE aerogel, exhibits the desired capabilities of self-powering and ultrasensitive high-temperature warning, making it well-suited for firefighters. The highly elastic and high-temperature-resistant PEDOT:PSS/SWCNT composite aerogels proposed in this work exhibit great potential in the fields of strain sensor, wide-range temperature heat harvesting, as well as self-powered high-temperature monitoring/warning in wearable electronics.

## Conclusion

In summary, ultralight weight, elastic, flame retardancy and high-temperature-resistant PEDOT:PSS/SWCNT composite TE aerogels are fabricated via a convenient solvent displacement, solution mixing and subsequent freeze-drying process, exhibiting a large Seebeck coefficient of 38.9 μV K^−1^ and a relatively low thermal conductivity of 0.074 W m^−1^ K^−1^. The aerogels reveal a remarkable enhancement in both electrical and thermal conductivities as the compressive strain increases, while the Seebeck coefficient can be well maintained, leading to a greatly enhanced power factor of 0.58 μW m^−1^ K^−2^ at the strain of 80%. Benefiting from the high elasticity, excellent TE performance and high-temperature resistance of the aerogels, the fabricated aerogel-based sensors demonstrate the capability to accurately detect pressure stimuli and achieve wide-range temperature monitoring. A flexible TE generator is subsequently fabricated by connecting 25 aerogels in series, generating a maximum output power of 400 μW at a high-temperature difference of 300 K. Notably, the testing or application temperatures of our fabricated TE aerogel and its TE generator are significantly higher than that of mostly reported aerogels and their generators. Moreover, the reported ultralight high-temperature-resistant TE aerogel can also serve as a self-powered wearable sensing glove for wide-range temperature detection and complex hand gestures recognition. The designed self-powered intelligent fire warning system, incorporated into firefighting cloths, enables highly sensitive and repeatable monitoring and alarm capabilities for high-temperature fire sources. With all these desirable properties, the elastic, flame retardancy and high-temperature-resistant aerogel holds promising applications in wide-range temperature heat harvesting, high-temperature monitoring/warning, and self-powered wearable electronics.

## Supplementary Information

Below is the link to the electronic supplementary material.

Supplementary file1 (PDF 1466 KB)

Supplementary file2 (MP4 3978 KB)

Supplementary file3 (MP4 27452 KB)

Supplementary file4 (MP4 23733 KB)

Supplementary file5 (MP4 2732 KB)
