# Supplementary material for: Harness High-Temperature Thermal Energy via Elastic Thermoelectric Aerogels
Source: Nanomicro Lett. 2024 Mar 11;16:151. doi: 10.1007/s40820-024-01370-z (PMC10928063; doi:10.1007/s40820-024-01370-z)
Supplement: Supplementary file 1 — Supplementary file1 (PDF 1466 KB) [file 40820_2024_1370_MOESM1_ESM.pdf]

Supporting Information for

Harness High-Temperature Thermal Energy via Elastic Thermoelectric Aerogels

Hongxiong Li[1, #], Zhaofu Ding[1, #], Quan Zhou[1], Jun Chen[2], Zhuoxin Liu[1], Chunyu Du[1], Lirong Liang[1, *], Guangming Chen[1, *]

[1] College of Materials Science and Engineering, Shenzhen University, Shenzhen 518055, P. R. China

[2] Department of Bioengineering, University of California, Los Angeles, CA 90095, United States

[#] Hongxiong Li and Zhaofu Ding contributed equally to this work.

*Corresponding authors. E-mail: lianglirong@szu.edu.cn (Lirong Liang); chengm@szu.edu.cn (Guangming Chen)

Supplementary Figures and Tables

Mechanical properties of composite aerogels with or without PEDOT:PSS

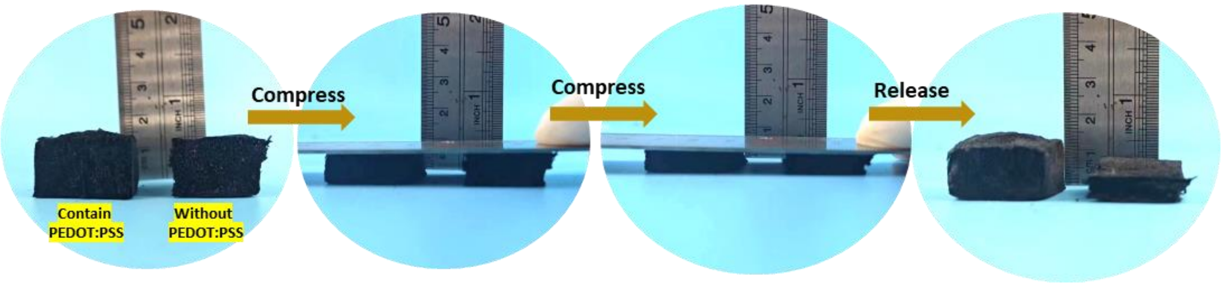

Fig. S1 Digital photos of the state of composite aerogel with and without PEDOT:PSS before and after compression

Figure S1 illustrates that the absence of PEDOT:PSS resulted in an aerogel that could not fully recover its original state after compression, indicating insufficient resilience. PEDOT:PSS plays a crucial role in the mechanical (elastic) and TE properties of the PEDOT:PSS /SWCNT composite aerogel. On one hand, the presence of PSS (polystyrene sulfonic acid) in PEDOT:PSS interacts with crosslinkers (3-glycidyloxypropyltrimethoxysilane, GOPS) and a small amount of carboxymethyl nanocellulose, which contributes to the improvement of mechanical properties. On the other hand, there is also a π-π interaction between the PEDOT molecular chain and SWCNT, promoting the enhancement of both TE and mechanical properties.

PTFE molds

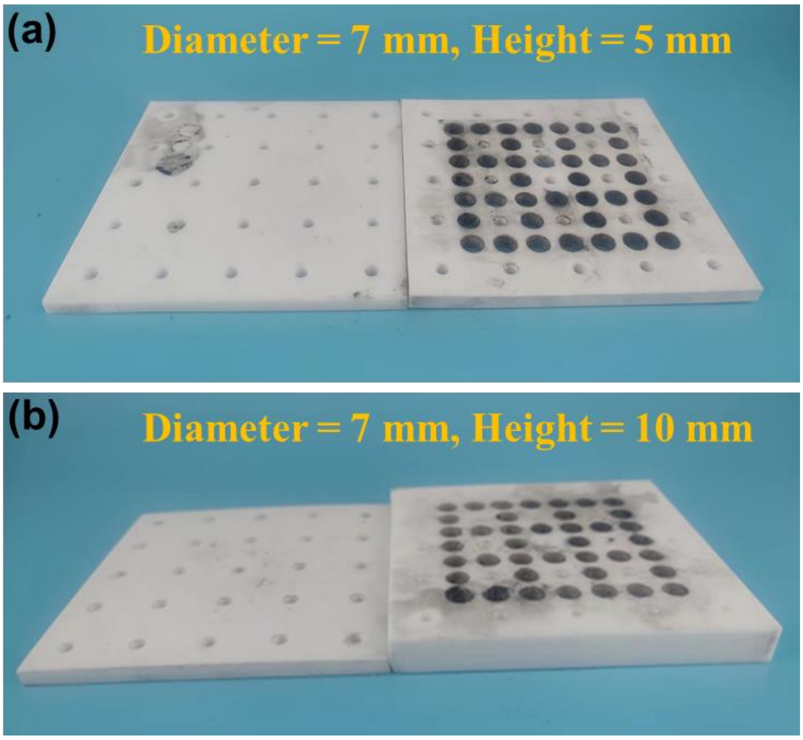

Fig. S2 Photographs showing the specific-sized PTFE molds: **a** Diameter is 7 mm and height is 5 mm. **b** Diameter is 7 mm and height is 10 mm

Self-established apparatus

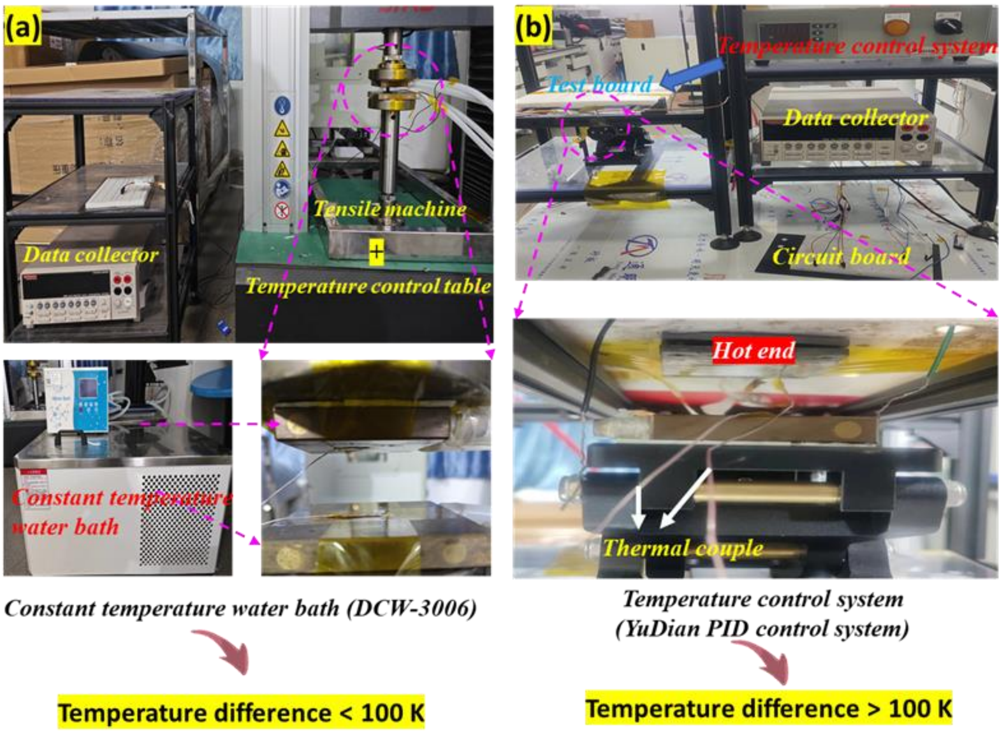

Fig. S3 a Photos showing the self-established apparatus for the measurement of the electrical conductivity and Seebeck coefficient at different compression strains. **b** Photographs of a self-established and temperature-controllable hot stage equipment to test the output performances of the aerogel-based TE generator

Mechanical stress-strain tests

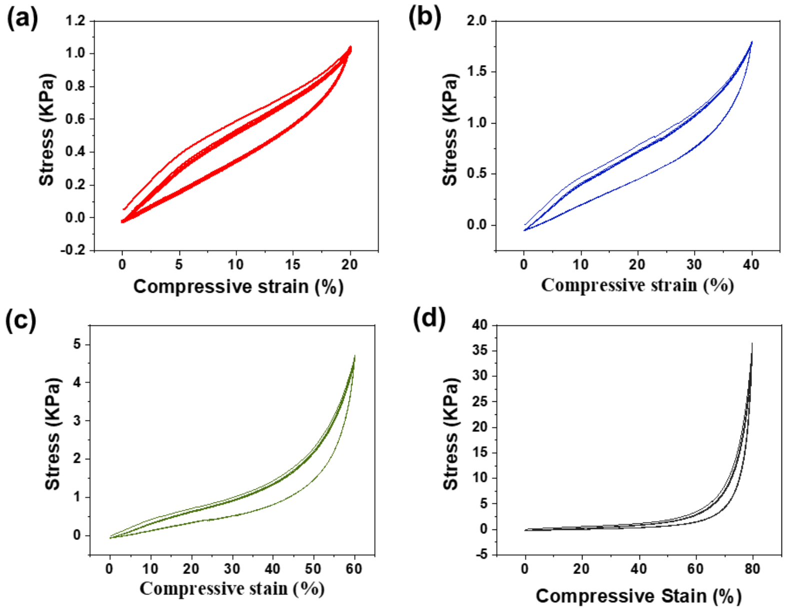

Fig. S4 The stress-strain curves of the composite aerogel were cyclically compressed 1–5 times under different strains: **a** 20%, **b** 40%, **c** 60% and **d** 80%

Thermal conductivity mesurement

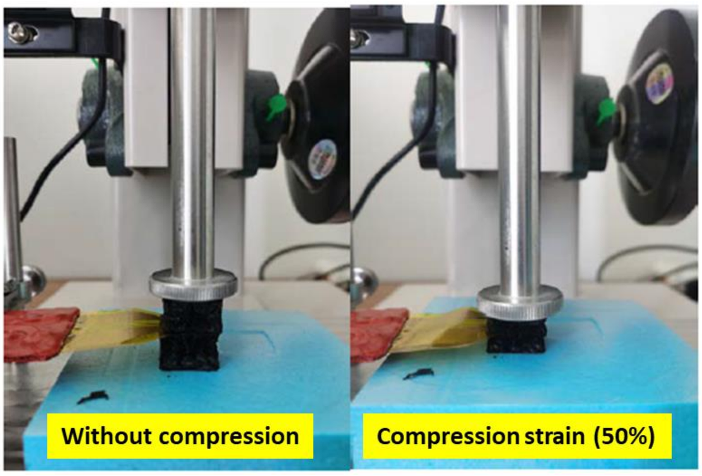

Fig. S5 Photos showing the thermal conductivity test of the aerogel

The thermal conductivity (κ) of the aerogel was measured using the Hot Disk thermal constant analyzer (Hot Disk TPS3500) based on the principle of transient plane source method. The testing process is illustrated in **Fig. S5**, where two aerogel samples with dimensions of approximately 15 mm × 15 mm × 10 mm (length × width × height) were tested for thermal conductivity in original state (without compression) and compressed state (50% compression). The probe is sandwiched between the two samples to form a sandwich structure for thermal conductivity testing (each state is tested twice to verify the accuracy of the results).

The testing results are presented in **Table S2**, and a screenshot of the testing results is shown in **Fig. S6**. This testing method provides information on thermal diffusion coefficient (α) and volumetric heat capacity (c_v). By measuring the mass and volume of the samples, the density (ρ) can be calculated. Finally, using the formula $\kappa = \rho \times c_v \times \alpha$, the thermal conductivity κ of the aerogel can be determined.

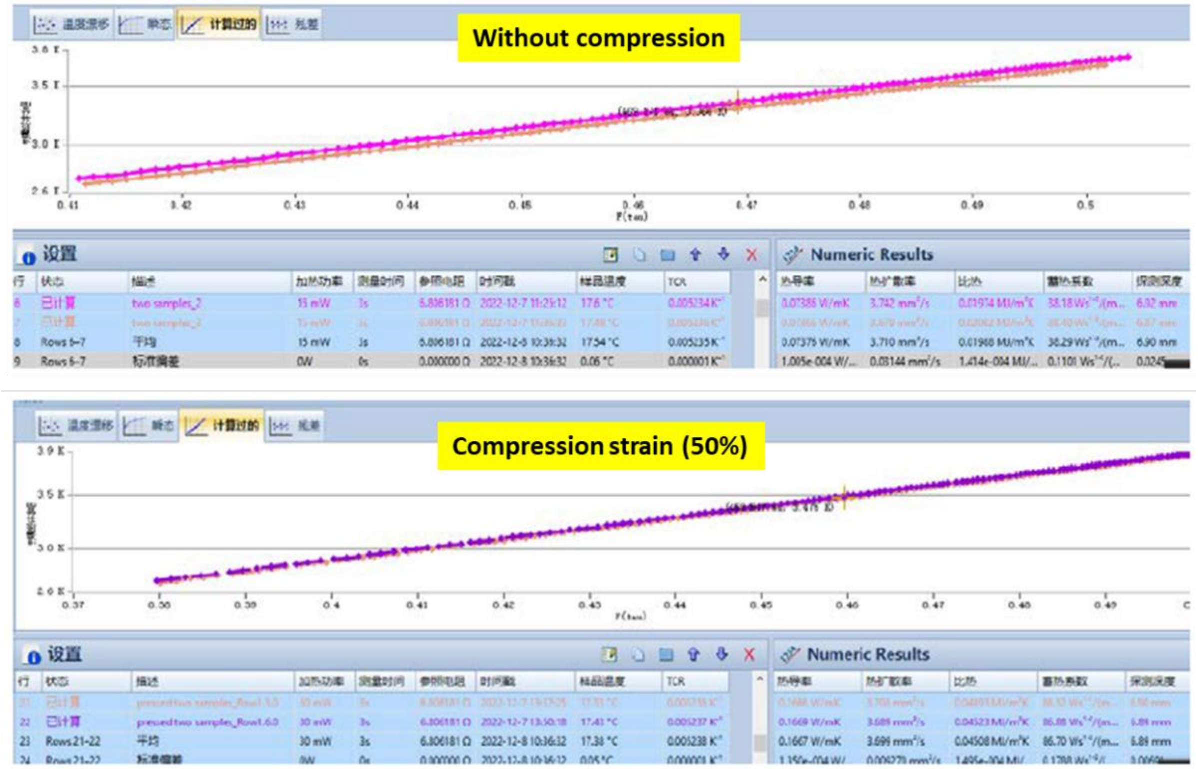

Fig. S6 Screenshots of thermal conductivity test results

High-temperature resistance of aerogel sensor

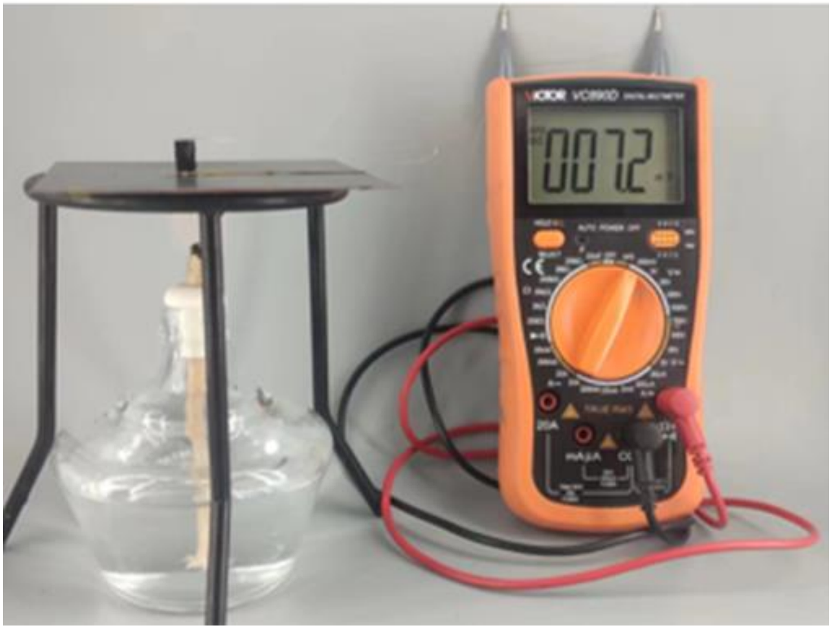

Fig. S7 Photograph showing the generated voltage of the aerogel sensor by harvesting alcohol lamp flame heat energy

Thermogravimetric analysis

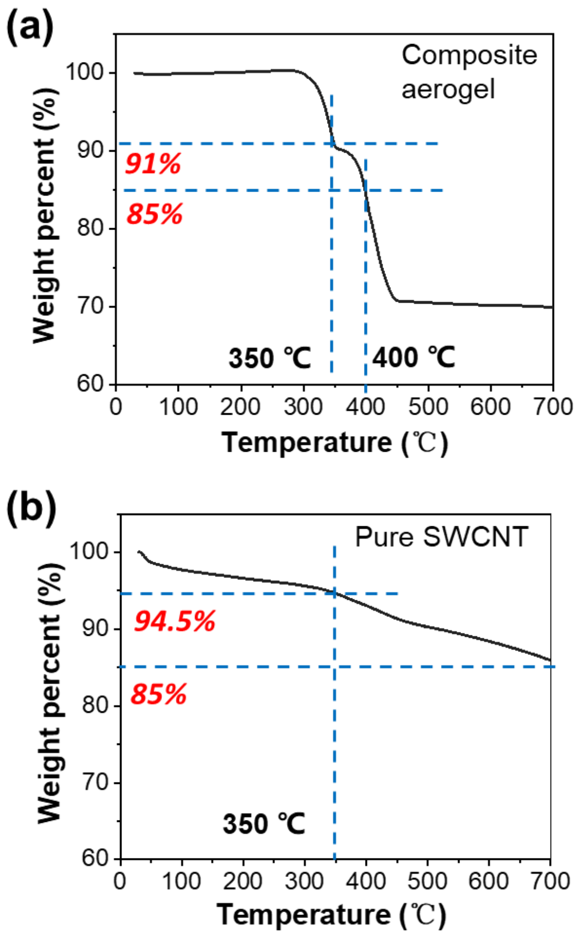

Fig. S8 a TGA curve of the PEDOT:PSS/SWCNT aerogel. **b** TGA curve of the pure SWCNT

Output power of the TE generator

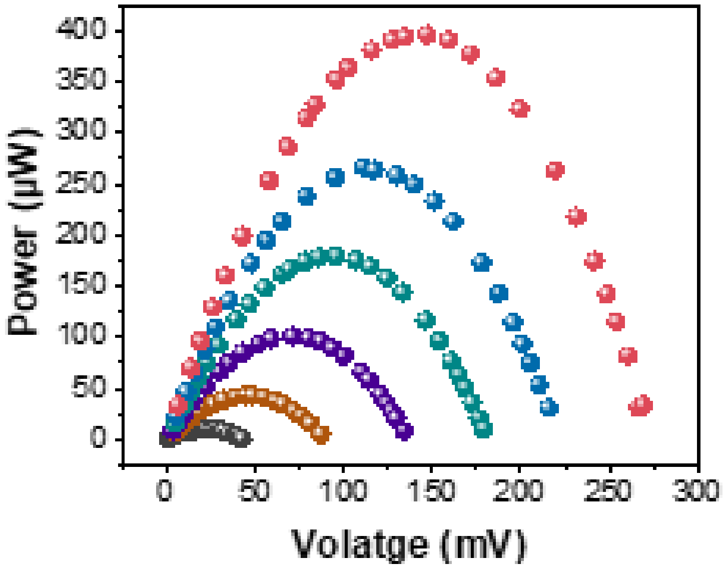

Fig. S9 The output power of the TE generator as a function of circuit voltage under different temperature differences (50–300 K)

Output voltage and current of the TE generator

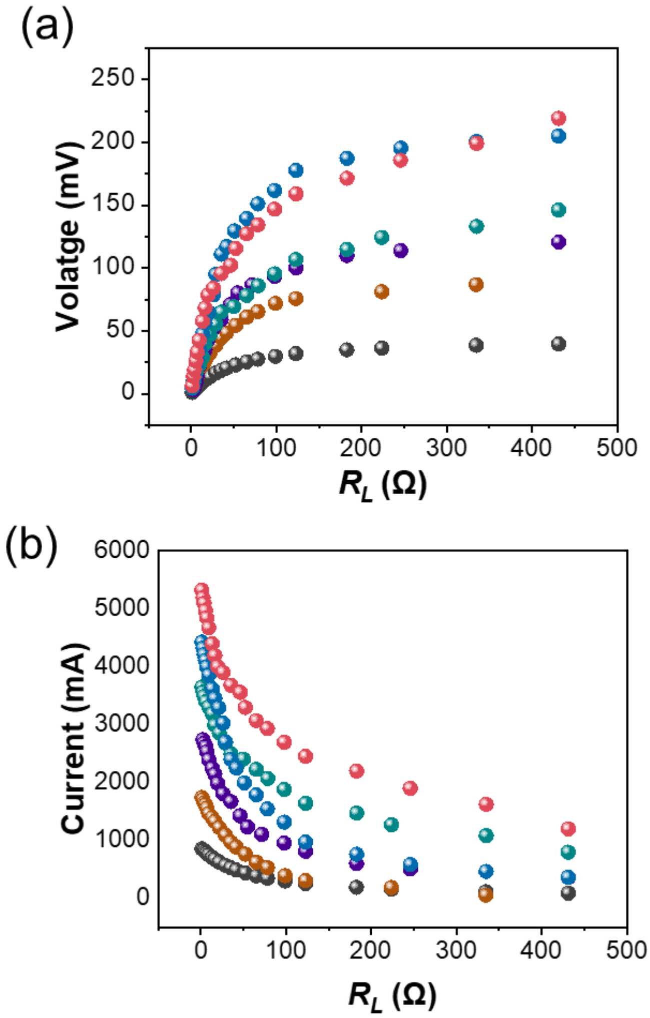

Fig. S10 The **a** output voltage and **b** circuit current as a function of load resistance under different temperature differences (50–300 K)

Internal resistance of the TE agenerator

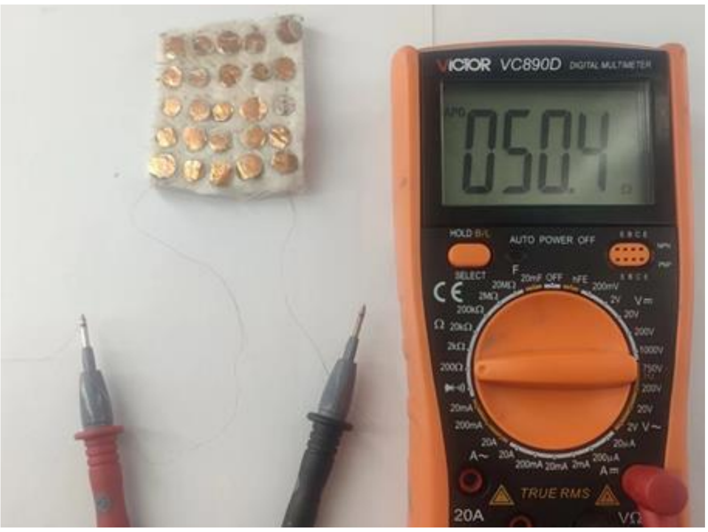

Fig. S11 The internal resistance of the aerogel-based TE generator

Various hand gestures

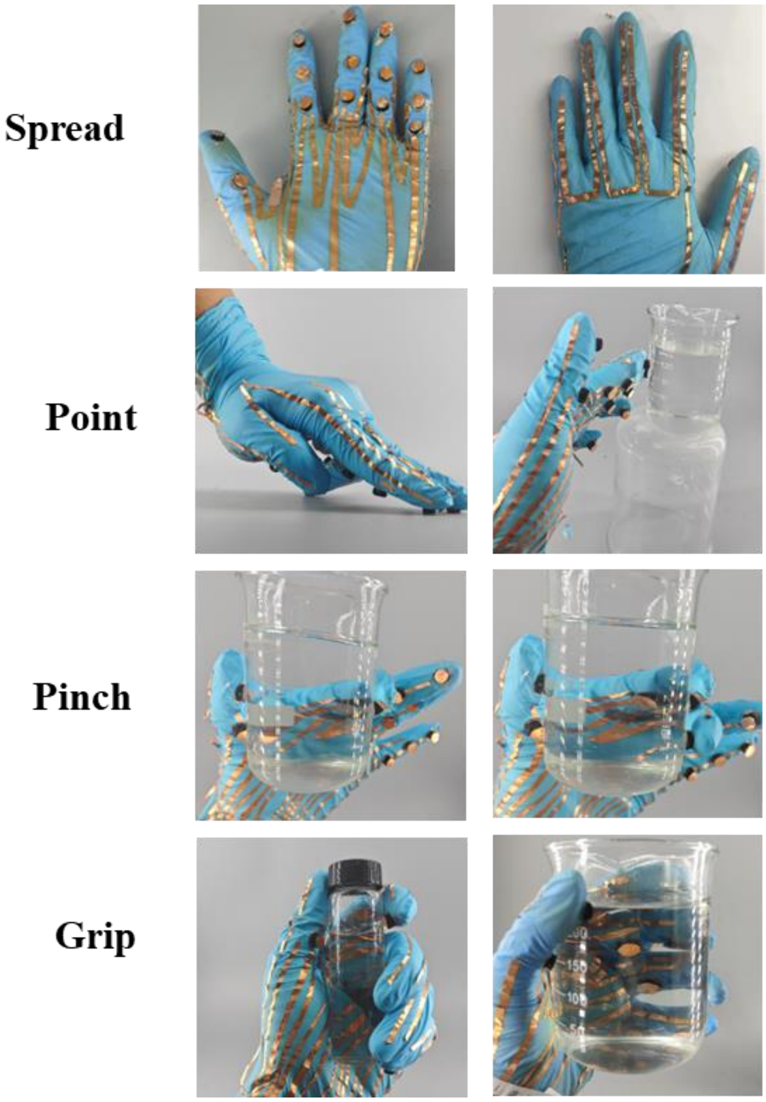

Fig. S12 Photographs showing the hand gestures of “Spread”, “Point”, “Pinch” and “Grip” when wearing the integrated aerogel-based sensor glove

Hand motion recognition

The integrated thermoelectric (TE) sensing glove can be used to hand motion recognition. It is well-known that the TE sensing glove, when in contact with an object, generate a voltage signal that is highly dependent on the temperature difference (ΔT) between the object and human finger heat.

Specially, three characteristic voltage signals (Knuckle 1, Knuckle 2 and Knuckle 9) are collected to elucidate the application of the integrated TE sensing glove for hand motion recognition (such as spread, point, pinch, and grip). Distinct voltage amplitudes in the three knuckle sensors enable the recognition of hand gestures. The "Spread" pattern is characterized by the relatively similar voltage amplitude in all three knuckles. The "Point" pattern is characterized by a high voltage amplitude in Knuckles 2 and low voltage amplitudes in Knuckle 1 and Knuckle 9. The "Pinch" pattern is characterized by a high voltage amplitude in Knuckle 1 and Knuckle 2, and a low voltage amplitude in Knuckle 9. The "Grip" pattern is characterized by a high voltage amplitude in Knuckle 2 and Knuckle 9, and a low voltage amplitude in Knuckle 1. It should be noted that the voltage amplitude is nearly same when applying the same ΔT.

Herein, three voltage parameters are distinguished in the recognition tests: the measured voltage amplitude (V_X), the maximum voltage (V_{max}), and the threshold voltage (V_{tX}). V_{max} refers to the highest voltage generated by the sensor under a certain temperature difference. V_{tX} is defined as a characteristic voltage for recognizing hand gestures, it can be calculated by Equation $V_{tX} = kV_{max}$, where subscript X represents the sensor name corresponding to the knuckle order (**Fig. 5a**), and k is the ratio of V_{tX} to V_{max} and can be adjusted artificially. For instance, V_{t1} represents the threshold voltage of Knuckle 1. The optimal *k* value is determined by recognition experiment.

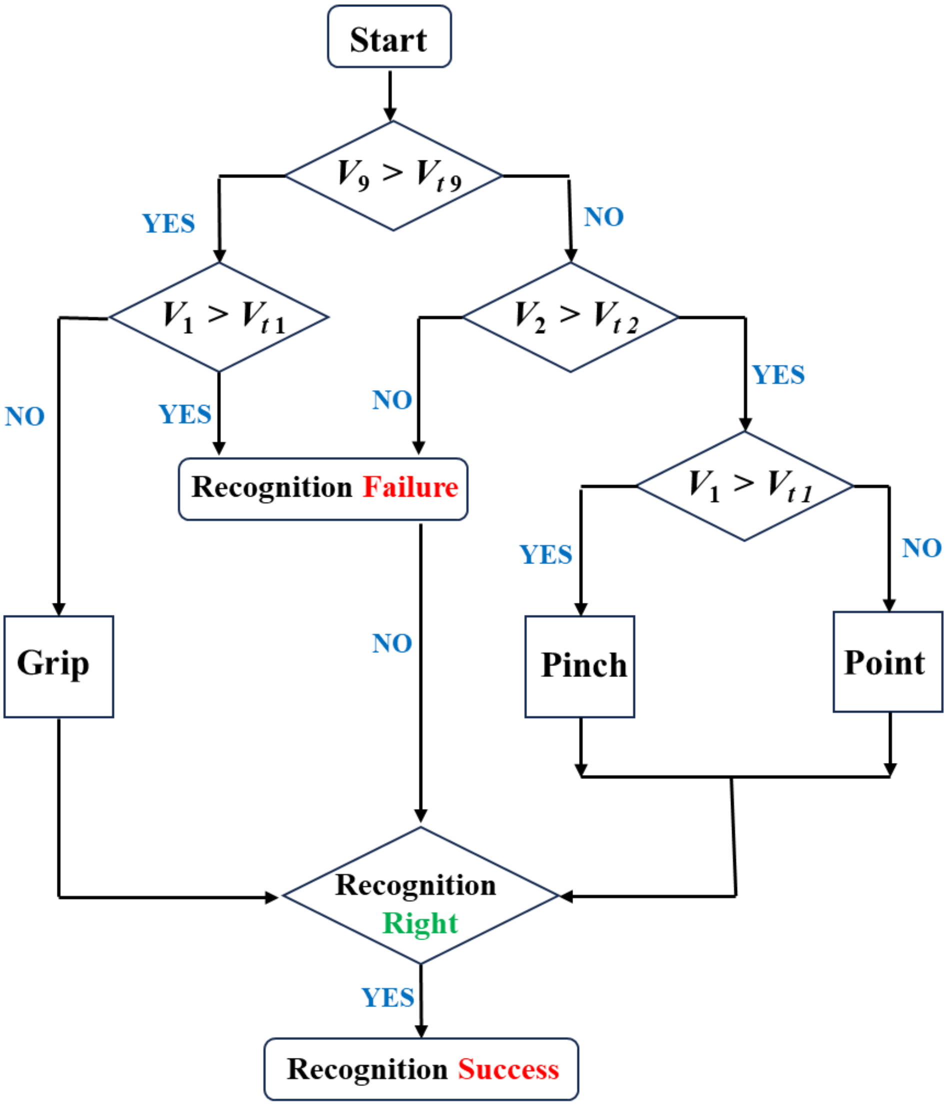

Fig. S13 The algorithm logic diagram in hand motions recognition experiment

The algorithm logic diagram for the recognition experiment is illustrated in **Fig. S13**. In the diagram, V_X represents the measured voltage in the recognition experiment, with subscript X denoting the sensor name corresponding to the knuckle order. For example, V_2 represents the measured voltage of Knuckle 2 in the recognition experiment. Combing the algorithm logic diagram and the set V_{tX}, we can accurately achieve hand motion recognition.

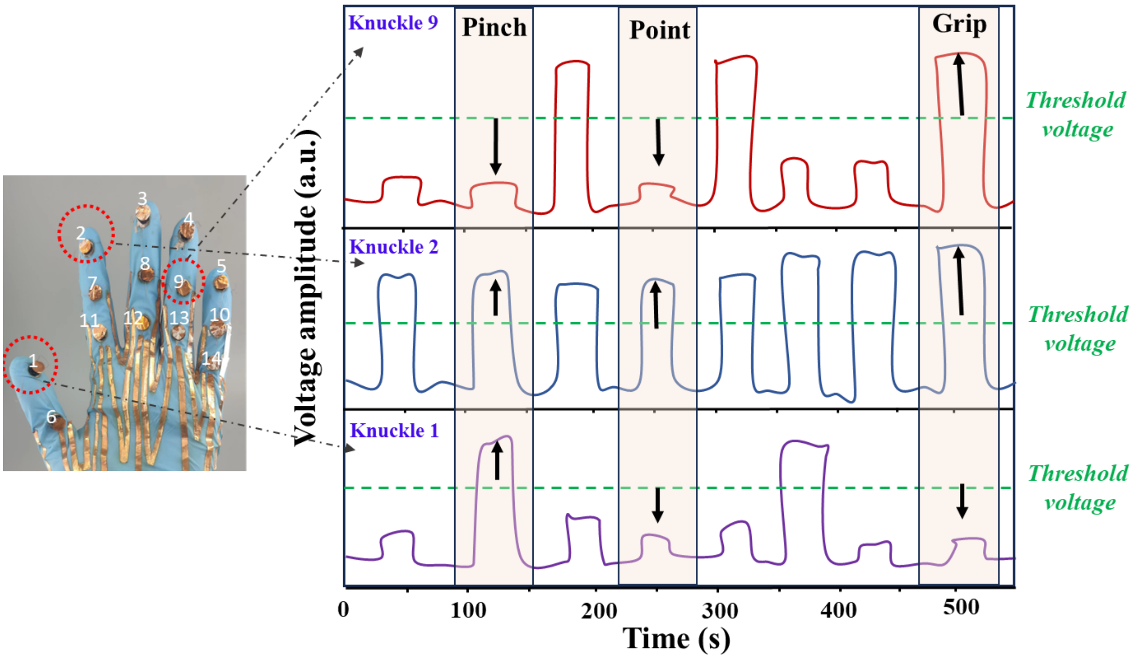

Fig. S14 The voltage amplitudes generated in three knuckles (knuckle 1, 2 and 9) during a series of random hand motions. The green dash line represents the "threshold voltage" with a *k* value of 0.55 in the recognition experiment

Based on previous empirical values [39], setting *k* is 0.55, the voltage amplitude variations at the three Knuckles are shown in **Fig. S14**. It can be observed that the hand gestures of "Point," "Pinch," and "Grip" exhibit distinct changes near the threshold voltage (V_{tX}), indicating its effective hand motions recognition capability.

Repeatable fire warning behavior

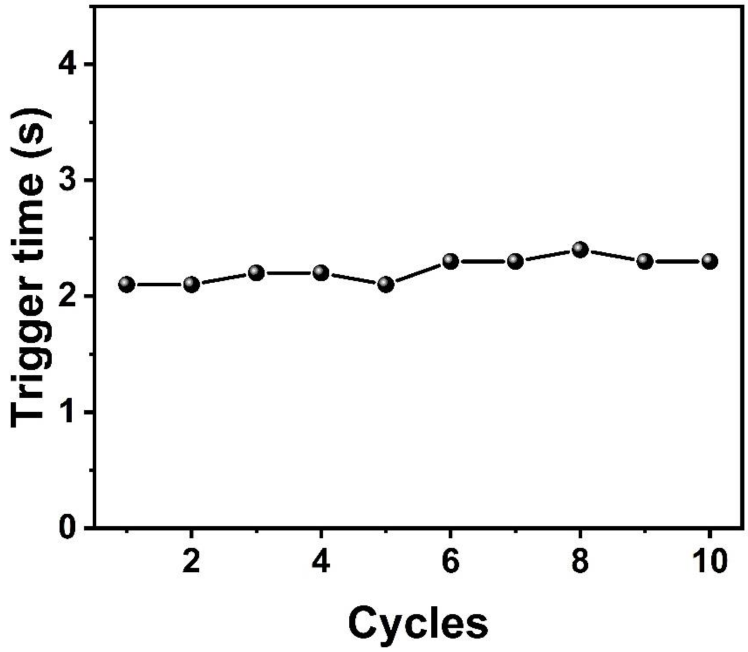

Fig. S15 The trigger times corresponding to each fire warning during these 10 cycles

The intelligent fire warning system

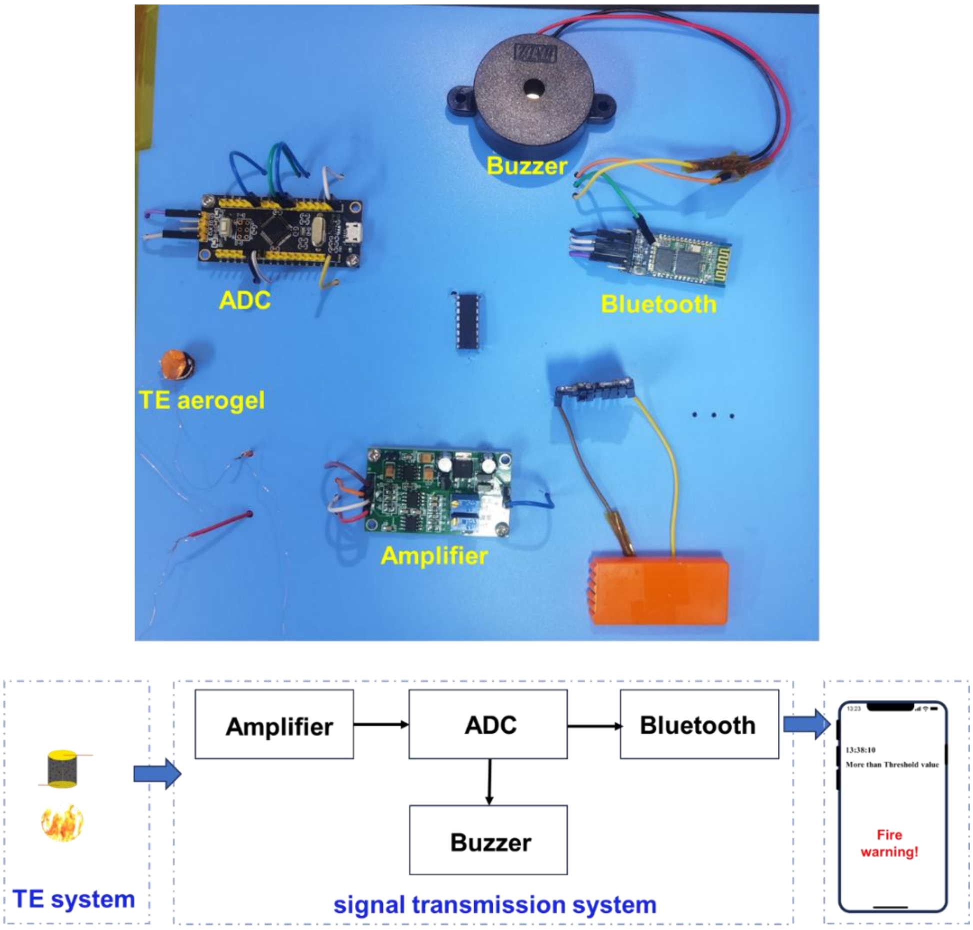

Fig. S16 The designed self-powered smart fire warning system

TE properties of composite aerogels with different PEDOT:PSS contents

Table S1 The TE properties of various PEDOT/SWCNT composite aerogels with different PEDOT:PSS contents

**Weight ration of PEDOT:PSS:SWCNT:NFC:GOPS**	***S*** **(μV K^{-1})**	***σ*** **(S cm^{-1})**	***PF*** **(μW m^{-1} K^{-2})**
0 : 136 : 15 : 150	29.36±0.5	0.1	0.008
15 : 136 : 15 : 150	39±0.3	1.5	0.23
45 : 136 : 15 : 150	37±0.6	0.5	0.06
68 : 136 : 15 : 150	30±0.5	0.73	0.07
136 : 136 : 15 : 150	34.7±0.3	0.02	0.001

Firstly, the content of SWCNT (136 mg) is fixed, and the content of NFC (15 mg) is determined to ensure the dispersion of SWCNT. The ratio of NFC to the crosslinker GOPS is

also fixed (NFC:GOPS = 1:10). The optimal mass ratio of PEDOT:PSS to SWCNT in composite aerogels was determined based on the desired mechanical and TE properties of the resulting aerogels, aiming to achieve excellent elasticity and TE performance. **Table S1** presents the results, it can be seen that a high PEDOT:PSS content significantly reduced the power factor (*PF*) of the composite aerogel, impacting its electrical conductivity (σ) or Seebeck coefficient (S). This is because the commercially PEDOT:PSS aqueous solution itself exhibits lower TE performance (σ = 0.1 S cm^{-1}, S = 15.5 μV K^{-1}). In this study, we did not carry out any additional performance modifications on PEDOT:PSS. Thus, a high PEDOT:PSS content would diminish the TE properties of the composite aerogel. Therefore, the optimal weight ratio of PEDOT:PSS to SWCNTs is 15:136. Combining the above points, the final optimal weight ratio of PEDOT:PSS:SWCNT:NFC:GOPS = 15:136:15:150.

Table S2 Thermal conductivity parameters obtained from tests of PEDOT/SWCNT composite aerogels in the original and compression states

Sample	Thermal conductivity (W m^{-1} K^{-1})	Thermal diffusion coefficient (mm^2 s^{-1})	Volume specific heat (MJ m^{-3} K^{-1})
Without compression (ε=0)	0.07386	3.742	0.01974
	0.07366	3.679	0.02002
Compress strain 50%	0.1666	3.708	0.04493
	0.1669	3.689	0.04523

TE performance comparison of various aerogels

Table S3 Comparison of the TE performance of various polymer or polymer/carbon nanoparticle composite aerogels

Constituent	σ (S cm^{-1})	S (μV k^{-1})	κ (W m^{-1} k^{-1})	PF (μW m^{-1} k^{-2})	ZT	Strain range	Tolerable temperature (°C)	Refs.
SWCNT– PU	0.028	37.05	0.055	3.84×10^{-3}	2.1×10^{-5}	10–80%	45	[19]
SWCNT– PU	0.073	38.91	0.08	11×10^{-3}	3.86×10^{-5}	10–80%	-	[19]
SWCNTs/Ag (42.56wt%)	9.2	54	0.077	2.863	0.011	-	-	[S1]
CNF/CNTs (25wt%)	0.029	15	0.038	6.525×10^{-4}	0.0547×10^{-8}	0–70%	-	[S2]
C-CNF/CNTs (5wt%)	0.43	25	0.08	0.0269	-	90%	60	[32]
CNT	0.135	26	0.05	0.09	9×10^{-4}	-	-	[S3]
CNT/PEDOT	502	31.37	0.12–0.17	49.3	0.087	30%	47	[31]
CNT/PEDOT	267.1	16.54	-	7.3	-	-	-	[31]
	512.6	16.54	-	15.4	-	-	-	[31]
CNT/PEI	98.06	−29.3	0.12	8.42	0.021	30%	-	[31]
PEDOT:PSS	0.000206	13	2.94×10^{-2}	3.47×10^{-8}	9×10^{-4}	-	-	[11]
PEDOT:PSS	2.35	17	2.94×10^{-2}	0.07	7×10^{-3}	-	-	[11]
PEDOT:PSS/SWCNTs	-	40.05	-	-	-	0–80%	60	[23]

PEDOT:PSS/SWCNTs	0.083	35.9	0.077	-	-	0–70%	60	[20]
MWCNT/RF	3.8	46	0.007	0.8	3.3×10^{-3}	-	-	[S4]
rGO/SWCNTs	6.66	29	1.5×10^{-2}	0.56	8.03×10^{-3}	0–40%	-	[S5]
CNT	1.23	18.2	-	0.04	-	-	-	[S6]
CNT (K)	1.1	−12.5	-	0.017	-	-	-	[S6]
CNT (I2)	2.97	20.5	-	0.125	-	-	-	[S6]
Graphene/MWCNT	0.52	58	0.056	0.175	-	-	-	[S7]
Graphene/C	3.25	82	0.064	2.185	-	-	-	[S8]
Graphene/PDMS		35.2	-	-	-	41 kPa	50	[34]
Graphene /TPU/CNFs	30.8	-	-	-	-	60 kPa	66	[35]
PANI/GO	92	37.5	0.2	13.2	1.02×10^{-2}	0–50%	-	[12]
rGO/GNP	-	-	0.17		-	-	77	[14]
Graphene/RF	3	80	0.06	1.92	-	-	-	[S9]
Graphene/C	3.25	82	0.064	2.185	-	-	-	[25]
PEDOT:Tos/SWCNTs	2.5	50	0.09	0.6	0.02	0–80%	75	[18]
BC/PEDOT/SWCNT	0.8	28.6	0.0322	0.3	-	-	-	[38]
PEDOT:PSS/CNF/PEI/CP	6.7	21	0.0449	2.95	1.88×10^{-6}	80%	37	[S10]

MoS2 /PEI	0.861	82	1.05	5.7	5.4×10^{-3}	30% stretch	-	[S10]
NbSe2/PEI	1.9	−8.5	0.423	0.137	1.36×10^{-4}	30% stretch	-	[24]
Graphene /PDMS	0.0845	49.2	-	0.188	-	50%	80	[S11]
PDMS/Ts/PPy/rGo	0.74	84.2	5.977	0.249	-	-	-	[17]
PEDOT:PSS/MWCNT/Ag	6.711	61.3	0.12	7.56×10^{-3}	-		-	[S12]
P3HT/PS	7.5×10^{-4}	3000	-	0.68	-	-	-	[33]
CrSi2/NFC-PEDOT:PSS	5.4	88	12.5	4			32	[37]
PEDOT:PSS/SWCNT	1.5	39.4	0.074	2.29	-	0	350	**This work**
	3.7	39.4	-	5.67	-	80%	350	

Output performance comparison of the aerogel-based TE generators

Table S4 Comparison of the output performance of aerogel-based TE generators

p-type	n-type	Units	ΔT (K)	U_{oc} (mV)	P_{max} (μW)	$P_{density}$ (μW cm^{-2})	Refs.
SWCNT/PU	SWCNT/$FeCl_3$	8 p-n	55	31	0.17	0.13	[19]
PEDOT:PSS/CNF/PEI/CP	/	1 p	16	0.3	-	-	[38]
MoS_2/PEI	$NbSe_2$/PEI	1 p-n	7	0.61	-	-	[S10]
MoS_2/PEI	$NbSe_2$/PEI	2 p-n	7	1.47	-	-	[S10]
MoS_2/PEI	$NbSe_2$/PEI	3 p-n	7	2.01	-	-	[S10]
PEDOT:PSS/SWCNTs/melamine	/	25 p	5	10	-	-	[20]
SWCNTs/Ag	/	1 p	52.8	0.8	-	-	[1]
BC/PEDOT/SWCNT	/	1 p	50	1.1	84	0.021	[18]
PEDOT:TOS/SWCNTs	/	10 p	60	28	1967	3.073	[25]
CNT/PEDOT:PSS	CNT/PEI	9 p-n	39.4	13.6	1900	0.094	[31]
C-CNF/CNTs	/	1 p	30	0.8	0.07	3.5×10^{-5}	[32]
PEDOT:PSS/SWCNT-PDMS	/	25 p	10	5.94			[23]
PEDOT:PSS/SWCNT	/	25 p	300	275	400	40	**This work**

Table S5 Comparison of the response time of various TE warning materials

System	Threshold (mV)	Response time (s)	Refs.
MXene/ANFs	0.1	Within 10	[1]
MXene/CCS	1	3.8	[2]
MXene/AgNWs/ANFs	1	1.6	[3]
PI/MXene	1	4.67	[4]
PI/MXene/Ag_2Se	1	4.03	[5]
MXene/TA/$CaCl_2$	4	Within 3	[6]
MXene/UPC/MMT	1	3.1	[7]
CNT/MXene/CNFs/MMT	1	1.43	[8]
**PEDOT:PSS/SWCNT**	**1**	**2.1**	**This work**

Supplementary References

[S1] X. Sun, J. Zhao, L. Zhao, J. Wu, Q. Li, Thermoelectric performance of conducting aerogels based on carbon nanotube/silver nanocomposites with ultralow thermal conductivity. RSC Adv. **6**(111), 109878–109884 (2016). https://doi.org/10.1039/c6ra17348a

[S2] R. Muthuraj, A. Sachan, M. Castro, J.-F. Feller, B. Seantier et al., Vapor and pressure sensors based on cellulose nanofibers and carbon nanotubes aerogel with thermoelectric properties. J. Renewable Mater. **6**(3), 277–287 (2018). https://doi.org/10.7569/jrm.2017.634182

[S3] X. Yang, J. Cui, K. Xue, Y. Fu, H. Li et al., Thermal conductivity and thermoelectric properties in 3D macroscopic pure carbon nanotube materials. Nanotechnol. Rev. **10**(1), 178–186 (2021). https://doi.org/10.1515/ntrev-2021-0013

[S4] L. Zhao, J. Zhao, X. Sun, Q. Li, J. Wu et al., Enhanced thermoelectric properties of hybridized conducting aerogels based on carbon nanotubes and pyrolyzed resorcinol–formaldehyde resin. Synt. Met. **205**, 64–69 (2015). https://doi.org/10.1016/j.synthmet.2015.03.036

[S5] D. Tan, J. Zhao, C. Gao, H. Wang, G. Chen et al., Carbon nanoparticle hybrid aerogels: 3D double-interconnected network porous microstructure, thermoelectric, and solvent-removal functions. ACS Appl. Mater. Interfaces **9**(26), 21820–21828 (2017). https://doi.org/10.1021/acsami.7b04938

[S6] J. Chen, X. Gui, Z. Wang, Z. Li, X. Rong et al., Superlow thermal conductivity 3D carbon nanotube network for thermoelectric applications. ACS Appl. Mater. Interfaces **4**(1), 81–86 (2011). https://doi.org/10.1021/am201330f

[S7] L. Zhao, X. Sun, Z. Lei, J. Zhao, J. Wu et al., Thermoelectric behavior of aerogels based on graphene and multi-walled carbon nanotube nanocomposites. Compos. B Eng. **83**, 317–322 (2015). https://doi.org/10.1016/j.compositesb.2015.08.063

[S8] Z. Lei, Y. Yan, J. Feng, J. Wu, G. Huang et al., Enhanced power factor within graphene

hybridized carbon aerogels. RSC Adv. **5**(33), 25650–25656 (2015). https://doi.org/10.1039/c5ra03198b

[S9] J. Liu, X. Chen, H. Yang, J. Tang, R. Miao et al., Gel-emulsion templated polymeric aerogels for solar-driven interfacial evaporation and electricity generation. Mater. Chem. Front. **5**(4), 1953–1961 (2021). https://doi.org/10.1039/d0qm00793e

[S10] D.X. Nghia, J.J. Baek, J.Y. Oh, T.I. Lee, Deformable thermoelectric sponge based on layer-by-layer self-assembled transition metal dichalcogenide nanosheets for powering electronic skin. Ceram. Int. **49**(6), 9307–9315 (2023). https://doi.org/10.1016/j.ceramint.2022.11.097

[S11] M. Xiang, Z. Yang, J. Chen, S. Zhou, W. Wei et al., Polymeric thermoelectric composites by polypyrrole and cheap reduced graphene oxide in towel-gourd sponge fibers. ACS Omega **5**(46), 29955–29962 (2020). https://doi.org/10.1021/acsomega.0c04356

[S12] N. Okada, K. Sato, M. Yokoo, E. Kodama, S. Kanehashi et al., Thermoelectric properties of poly(3-hexylthiophene) nanofiber aerogels with a giant seebeck coefficient. ACS Appl. Polym. Mater. **3**(1), 455–463 (2020). https://doi.org/10.1021/acsapm.0c01185
